# Spatio-temporal drought monitoring in the Megech–Dirma watershed, Ethiopia, using Google Earth engine

**DOI:** 10.1038/s41598-025-29537-7

**Published:** 2025-11-26

**Authors:** Tade Mule Asrade, Getu Molla Yigzaw

**Affiliations:** 1 Department of Hydraulic and Water Resources Engineering, Debre Markos University, P.O. Box: 269, Debre Markos, Ethiopia; 2 Department of Information Technology, Debre Tabor University, Debre Tabor, Ethiopia

**Keywords:** Drought indices, Drought monitoring, Google earth engine (GEE), Megech–Dirma watershed, MODIS, Hydrology, Natural hazards, Engineering

## Abstract

Drought is a recurrent hazard with significant ecological and socioeconomic impacts, requiring continuous monitoring and assessment. This study evaluates the occurrence and spatial-temporal distribution of drought in the Megech–Dirma Watershed, Ethiopia, over the period 2001–2022. Agricultural drought indices Vegetation Condition Index (VCI), Temperature Condition Index (TCI), Vegetation Health Index (VHI) and the meteorological Standardized Precipitation Index (SPI) at 3-month temporal scales were analyzed using the Google Earth Engine platform. The time-series analysis revealed minimum VCI values, indicating severe drought, in 2009 and 2010, while moderate to no drought conditions dominated during 2001 to 2009 and 2010 to 2021. Moderate droughts also showed a declining trend in 2009 and 2010. The TCI series identified frequent extreme drought events in 2002, 2003, 2004, 2009, 2013, 2016, and 2019, with severe drought recorded in 2006, 2007, 2010, 2014, 2015, 2017, 2021, and 2022. Mild to moderate droughts occurred in 2001, 2002, 2008, 2018, 2020, and 2022. Minimum VHI values, also indicating severe drought, were observed in 2002, 2003, 2004, 2009, 2012, 2013, and 2016, while 2001, 2006, 2007, 2010, 2014, 2015, and 2017 to 2022 were dominated by mild to moderate drought conditions. SPI revealed moderate drought conditions, showing weak correlations with the vegetation-based indices (correlation coefficients: -0.08 with TCI, -0.04 with VCI, and − 0.06 with VHI). Drought severity increased spatially in the watershed’s northern regions. Of the total area, 9.63% experienced extreme drought, 20.95% severe drought, 29.12% moderate drought, 26.94% mild drought, and 13.36% no drought. Drought hazard maps revealed extreme and severe drought concentrated in the northern and southern parts, whereas moderate, mild, and no drought conditions were distributed across the southwest, east, and central regions. These findings highlight the spatial-temporal complexity of drought in the watershed and provide a robust basis for drought monitoring, water resource management, and ecological protection. The results emphasize the importance of continuous observation, proactive measures, and adaptive strategies to mitigate the impacts of drought.

## Introduction

Climate change is one of the greatest challenges facing humanity and natural ecosystems in the twenty-first century^1,2^. Global mean temperatures have increased by about 1.1 °C since the late nineteenth century, increasing the frequency and intensity of weather and climatic extremes such heatwaves, floods, and droughts^3,4^. According to the IPCC report, rising global temperatures and sea-level rise are intensifying the frequency and severity of extreme events, including droughts and floods^1,5^. Because natural systems are highly interconnected, a comprehensive scientific assessment remains essential for understanding the underlying causes and impacts of these changes.

Globally, drought has affected more people over the past five decades than any other natural hazard, undermining agricultural productivity, degrading ecosystems, and threatening food and water security^6,7^. Each year, an estimated 55 million people are directly affected by drought worldwide^8^. The consequences are particularly severe in developing regions that depend heavily on rain-fed agriculture and have limited adaptive capacity.

Africa, the second most populous continent in the world, is particularly vulnerable to climate change because of its high levels of exposure and low capacity for adaptation^1,9^. According to the IPCC^10^, the population living in African drylands is projected to double by 2050, with the number of people exposed to drought risk under a 2 °C warming scenario estimated at 974 million to 1.27 billion. Urban expansion is also expected to increase dramatically in arid and semi-arid regions by 180% in southern Africa, 300% in North Africa, and 700% in mid-latitude Africa by 2030^11^. Such rapid demographic and climatic changes will likely exacerbate water scarcity and land degradation across the continent.

East Africa is recognized as one of the world’s hotspots for climate vulnerability, where regular floods and droughts seriously damage agricultural systems and livelihoods^12,13^. Agriculture remains the backbone of the region’s economy, supporting more than 80% of the population and contributing about 42% of GDP^14^. However, the dominance of smallholder farming and the reliance on seasonal rainfall make the region extremely sensitive to rainfall variability and prolonged dry spells^15^. In recent years, the Horn of Africa has experienced some of its most severe droughts on record. For instance, the prolonged drought of 2022 affected approximately 36.4 million people across Ethiopia, Somalia, and Kenya^16^.

Over the past 50 years, Ethiopia has experienced frequent droughts with varying geographic and temporal intensity. The eastern, southeastern, and Rift Valley regions are particularly vulnerable^17^. Ethiopia is among the most drought-prone countries in Africa, where hydro-climatic variability has intensified extremes of precipitation and temperature, resulting in recurrent droughts^18^. The Somali Region, eastern Oromia, and the Upper Blue Nile basin including northern Tigray, parts of Amhara (South Wollo, North Wollo, and South Gondar), and Afar are especially drought-prone^19^. In recent decades, both meteorological and agricultural droughts have occurred almost every two years^20^, causing devastating impacts on agriculture, ecosystems, water resources, energy, transportation, food security, and livelihoods.

The Amhara National Regional State (ANRS), especially the dry and semi-arid regions, is one of the most severely affected locations. According to the 2023 report of the ANRS Disaster Risk Management and Food Security Commission (DRMFSC), drought events have a significant impact on the Abay and Tekeze river basin. Within these specific basins, the most severely impacted areas were the north Gondar, central Gondar, and Waghmira zones^21^.

The Megech–Dirma watershed, which lies within these drought-prone areas, is the focus of this study. Communities in this watershed primarily rely on rain-fed agriculture, making them highly vulnerable to seasonal droughts that result from erratic rainfall and rising temperature variability. Seasonal droughts are a long-standing challenge across Ethiopia, particularly in regions such as the Somali region, eastern Oromia, the Upper Blue Nile (UBN) basin, Northern Tigray, South and North Wollo, South Gondar, and Afar^16,19,22,23^. Historical analyses confirm the persistence of these events: Taye et al. reported that severe or extreme meteorological droughts occurred 7–11% of the time in UBN highlands, midlands, and lowlands over the last 38 years^24^, and Wubneh et al. documented significant drought spells in the basin in 1978/79, 1984/85, 1994/95, and 2003/04^19^.

Understanding drought characteristics is a critical step toward effective management. There is no single, widely recognized description for droughts because they are diverse and multidimensional. While they can be broadly classified as meteorological^12^, agricultural^25^, hydrological^19^, or socio-economic, their interactions are not straightforward^26^. Agricultural and hydrological droughts do not always coincide with meteorological droughts, as they are influenced by factors such as soil properties and watershed characteristics, which affect water storage and movement in soil, surface water, and groundwater^26^. Although they may not always coincide, meteorological and agricultural droughts are frequently associated in Ethiopia’s rainfed agricultural areas. Soil moisture at any given time depends not only on current precipitation but also on previous rainfall, soil type, and temperature.

Over the past few decades, several methods have been developed to monitor and statistically characterize drought, including both standardized and non-standardized indices applied in agriculture, hydrology, and meteorology^27^. Traditionally, drought monitoring relied on ground-based measurements from weather stations, with indices such as the Palmer Drought Severity Index (PDSI)^27^, the Standardized Precipitation Index (SPI)^27^, and the Standardized Precipitation Evapotranspiration Index (SPEI)^27^. However, many indices require long-term datasets or complex inputs and may not be universally applicable in data-scarce regions. The World Meteorological Organization (WMO) recommends the SPI as a standard for meteorological drought monitoring due to its simplicity, robustness, and flexibility across timescales^28^. Several studies, including Aksoy and Sertel^29^, have confirmed the reliability of SPI in diverse climatic regions, including South Africa^30^, China^31^, India^32^, Romania^33^, Ethiopia^34^, Germany^35^, Turkey^36^, Australia^37^, and Spain^38^. Consequently, the SPI was adopted in this study for meteorological drought assessment.

Traditional approaches for assessing and monitoring drought rely primarily on in situ precipitation records, which are generally accurate but often limited by data scarcity, uneven spatial distribution, and short temporal coverage^39^. Advances in remote sensing (RS) and Earth observation technologies, including NASA’s Landsat series launched in 1972, have transformed drought monitoring^40^. RS sensors provide meteorological and contextual data, including changes in plant health and water availability^41^. When combined with Geographic Information Systems (GIS), RS enables continuous monitoring across broad areas, addressing data scarcity issues in regions such as Ethiopia^42^.

Several RS-based drought indices have been developed, including the Normalized Difference Vegetation Index (NDVI)^43^, Vegetation Condition Index (TCI)^44^, (VCI)^45^, Temperature Condition Index, and Vegetation Health Index (VHI)^46^. TCI identifies vegetation stress from high temperatures, VCI detects changes in vegetation from unfavorable to favorable circumstances, and VHI integrates TCI and VCI to evaluate the overall health of the vegetation^47^.

Recently, hydrometeorological applications have made extensive use of Google Earth Engine (GEE), a cloud-based geospatial platform^48^. Since its introduction in 2010, GEE capabilities have been tested in a variety of applications, including vegetation mapping and monitoring^49^, land use/land cover change mapping^50^, and flood mapping^51^. Its cloud-computing infrastructure allows rapid processing of large geospatial and time-series datasets without local downloads^52^. GEE makes spatiotemporal analysis easier by lowering computing barriers, facilitating scalable, repeatable workflows for drought monitoring, and offering interactive tools^53^.

Several studies have assessed drought conditions in Ethiopia using vegetation- and rainfall-based indices. For instance, Wassie et al. used MODIS-based indices (NDVI anomaly, VCI, TCI, and VHI) to monitor agricultural droughts in North Wollo and determine years with severe drought from 2000 to 2019^23^. Enyew and Wassie^54^ extended this line of research in the Menna watershed, combining MODIS vegetation indices, CHIRPS rainfall, and CPC temperature data to reveal recurrent droughts and highlight rainfall variability as a major driver of vulnerability. Similarly, Tesfamariam et al. reported spatiotemporal variations in drought across the Rift Valley Lake Basin using SPI^55^, while Haile et al. employed SPEI and the Aridity Index to demonstrate ongoing drought events from 1988 to 2016 across multiple watersheds^56^. Despite these important contributions, most prior works examined either agricultural or meteorological drought in isolation, often at seasonal or annual scales, with limited attention to monthly variability, spatial autocorrelation, or comparative index reliability.

Unlike previous studies, this research integrates both agricultural (VCI, TCI, VHI) and meteorological (SPI) drought indices to generate spatial distributed drought hazard maps for the Megech–Dirma watershed. Meteorological drought, represented by SPI, is based solely on precipitation anomalies and the duration of dry periods. In contrast, agricultural drought is more complex, as it links meteorological conditions to crop stress, considering soil water deficits, evapotranspiration, and the variable susceptibility of crops at different growth stages. Indices such as VCI, TCI, and VHI are particularly effective in detecting agricultural drought by incorporating vegetation vigor, canopy temperature, and soil moisture stress. By integrating these indices, our study provides a more comprehensive and reliable assessment of drought dynamics. This approach reduces the bias of relying on a single indicator, captures both short-term meteorological variations and long-term vegetation stress, and highlights drought impacts across time, space, and sectors (agriculture, water, and ecosystems). Such integration enhances the accuracy of drought monitoring and improves its relevance for decision-making and mitigation strategies. This is also consistent with earlier research^20,57,58^, which demonstrated that combining indices leads to more nuanced drought assessments and better management outcomes.

Key innovations include mapping intra-seasonal drought dynamics, identifying hotspot clusters through spatial autocorrelation, evaluating inter-index relationships using PCA, and producing multi-index hazard zoning maps to support drought management. Implemented on Google Earth Engine (GEE), the methodology is scalable, reproducible, and applicable to other data-scarce basins.

## Materials and methods

### Study area

**Fig. 1:**
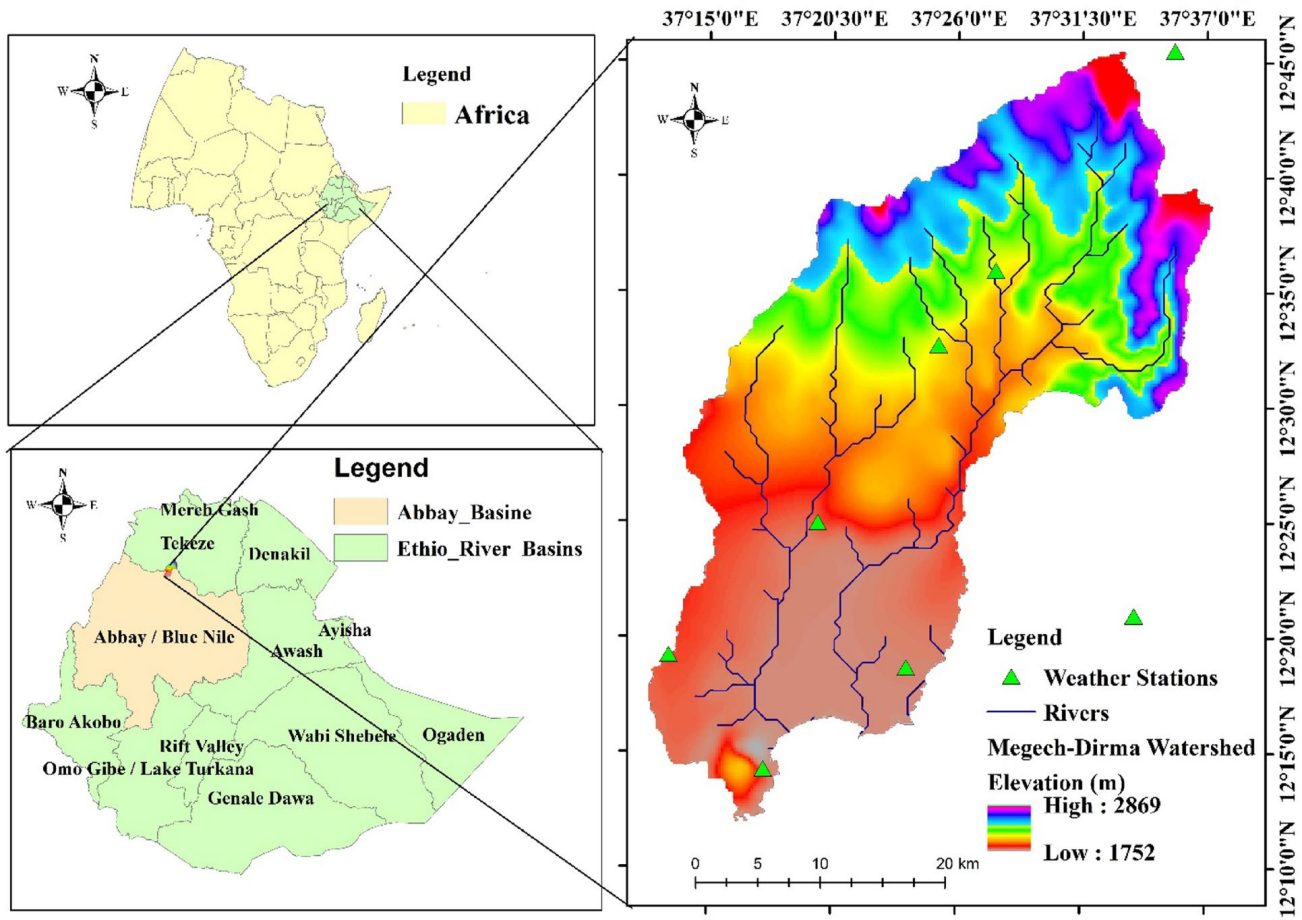
Location map of Megech–Dirma watershed.

Ethiopia’s Blue Nile basin has two major sub-basins: the Megech and Dirma watersheds. Their catchment area is roughly 1280.83 km², and they rise just from the highlands of Lay Armachiho and Gondar City. The elevation of the catchment ranges from 1755 to 2985 m above sea level. The Megech-Dirma rivers are located between latitudes 12°20′39″ N and 12°40′39″ N and longitudes 37°10′30″ E and 37°31′30″ E (Fig. 1). Beginning in the Layarmachiho and Wogera mountains, the Megech and Dirma rivers travel southeast via the Dembia floodplain and Gondar Zuria before entering Lake Tana. During the rainy season, the Megech and Dirma Rivers run quickly, and the sediments carried by the floods are mostly silt, stones, gravel, and sand^59^, which is characteristic of a mountainous stream. The average annual precipitation is approximately 1,050 mm, with the upper portion receiving 1,100 mm and the lower portion receiving approximately 1,000 mm. The temperature is between 11.5 and 30 degrees Celsius. Minimum temperatures range from 11.5 °C in January to 15.6 °C in April and May, while high temperatures range from 23 °C in July to 30 °C in March. There are currently six different types of land cover in the Megech-Dirma watershed: cultivated land, bare land, grassland, shrubland, urban, and plantation forests. The primary soil types found in the Megech-Dirma watershed were Eutric Leptosols, Eutric Vertisols, Urban, Chromic Luvisols, and Haplic Nitosols, in accordance with the FAO soil classification system. According to Habtu and Jayappa^59^, watershed areas have a high population density. This affects resource bases in several ways, including agricultural property, residential development, and deforestation. For many years, farmers in traditionally irrigated agriculture have used watersheds. Maps were created using ArcGIS software (version 10.4; Esri, Redlands, CA, USA; https://www.esri.com*).*

### Datasets and sources

#### CHIRPS precipitation data

For precipitation data, we used the Climate Hazards Group InfraRed Precipitation with Stations (CHIRPS v2), developed by the US Geological Survey (USGS) and the Climate Hazards Group at the University of California^23^. CHIRPS is a new rainfall product that provides quasi-global precipitation data at a 0.05° (~ 5 km) resolution by combining in situ station measurements with estimations from satellites^54^. Compared to other widely used products such as TRMM (25 km) and GPM (11 km), CHIRPS offers higher spatial and temporal detail, making it particularly robust in East Africa, including Ethiopia^23,54,60^. This high-resolution dataset is widely recognized as suitable for drought monitoring in data-scarce regions, where accurate rainfall estimates are essential for agricultural planning, drought assessment, and water resource management^61^.

This study utilized CHIRPSv2 rainfall data from 2001 to 2022 to analyze climate extreme indices. To evaluate its performance, previously published work^21^ compared CHIRPSv2 with ground-based rainfall records from seven weather stations across seasonal and annual timescales, demonstrating good agreement between the two datasets. Similar findings have been reported by several studies recommending CHIRPS for regional climate studies in Ethiopia^16,23^ as well as in other regions globally^53,62^. Additional validation studies in Ethiopia^17,23,54^ have also confirmed CHIRPSv2 as a reliable alternative to observed rainfall measurements.

Furthermore, the Standardized Precipitation Index (SPI) was derived from CHIRPS rainfall data, with the 3-month SPI (SPI-3) selected to capture short-term rainfall variability closely linked to agricultural drought conditions. Overall, evidence from both this study and previous research indicates that CHIRPSv2 serves as a robust replacement for gauge-based rainfall data in Ethiopia and similar data-scarce environments.

#### MODIS data

The Moderate Resolution Imaging Spectroradiometer (MODIS), a medium-resolution imaging spectrometer onboard NASA’s Terra and Aqua satellites, is a core component of the U.S. Earth Observing System (EOS)^23^. MODIS captures electromagnetic radiation across multiple spectral bands, enabling global monitoring of ecological, meteorological, and hydrological processes. Data products are available at spatial resolutions of 250 m, 500 m, 1 km, and a global 0.05° grid, with temporal resolutions ranging from daily to seasonal^61^. Aqua, launched in 2002, crosses the equator from south to north in the afternoon, while Terra, launched in 1999, travels from north to south in the morning^27^.

In this study, Terra MODIS data were used due to their longer temporal coverage and reliability for drought monitoring. The MOD13Q1 Version 6.1 product, a Level-3 dataset with a temporal resolution of 16 days and a spatial resolution of 250 m, was specifically used to generate vegetation indices. Normalized Difference Vegetation Index (NDVI) and Land Surface Temperature (LST) were taken from MODIS and then scaled to calculate the Vegetation Condition Index (VCI) and Temperature Condition Index (TCI). These indices were applied to characterize vegetation health and thermal stress, which are essential for assessing agricultural drought^16^. The dataset, covering the period 2001–2022, was accessed through the Google Earth Engine (GEE) platform.

MODIS was chosen because of its higher spatial resolution (250 m–1 km), broader spectral range, improved atmospheric correction, and accurate geo-location compared to its predecessor, the Advanced Very High Resolution Radiometer (AVHRR)^16,23^. These advantages have established MODIS as a reliable and widely used data source for agricultural drought monitoring and other environmental studies^16,23,62^.

### Data analysis methods

The Megech–Dirma watershed’s drought conditions from 2001 to 2022 were evaluated using the meteorological drought indices Standardized Precipitation Index (SPI) and agricultural drought indices, including the Vegetation Condition Index (VCI), Temperature Condition Index (TCI), and Vegetation Health Index (VHI). All datasets were processed using the Google Earth Engine (GEE) JavaScript code editor, which facilitates efficient retrieval, analysis, and export of satellite imagery.

NDVI and VCI were derived from MOD13Q1, while LST was obtained from MODIS11A2 products, and SPI was computed from CHIRPS precipitation data. All datasets were processed in the Google Earth Engine (GEE) platform, which enabled efficient retrieval, filtering, aggregation, and export of satellite imagery. Missing or null pixels in satellite products were handled using GEE’s masking and interpolation functions, and monthly aggregation minimized their impact on the indices.

To ensure comparability across datasets with different spatial resolutions (NDVI/VCI ~ 250 m, LST 1 km, CHIRPS ~ 5 km) and temporal frequencies (NDVI 16-day, LST daily, CHIRPS daily), all data were harmonized both temporally and spatially. Daily and 16-day data were aggregated to monthly values, NDVI/VCI were resampled to 1 km to match LST for VHI computation, and finally all indices were aggregated to the CHIRPS grid (~ 5 km) for direct comparison with SPI. Mean aggregation and re-projection preserved the integrity of each dataset while enabling robust correlation and spatial analysis. Core GEE functions (Map, filterDate, reduce, clip, expression, and Export) were used throughout to calculate drought indices and generate monthly drought severity layers, exported in GeoTIFF format with standardized projection and metadata.

#### Meteorological drought assessment methods

##### Standardized precipitation index

The Standardized Precipitation Index (SPI) is a widely used meteorological drought indicator that quantifies precipitation deficits relative to long-term averages^63^. It is recommended by the World Meteorological Organization (WMO) as a global standard due to its simplicity, robustness, and ability to assess drought across multiple timescales^28^. Over the past two decades, SPI has been extensively applied in drought research worldwide^64^. The SPI is calculated by fitting long-term precipitation records to a probability distribution, which is then transformed into a standardized normal distribution with a mean of zero^65^. Conditions that are wetter than normal are indicated by positive SPI values, whereas conditions that are drier than normal are indicated by negative values. Drought onset is generally defined when SPI ≤ − 1.0 and ends when SPI returns to positive values^66^. The SPI is computed by dividing the historical standard deviation of pixel i during timeframe j over n years, which is provided by Eq. 1, by the precipitation of pixel i during timeframe j of year k minus the historical mean of pixel i during timeframe j over n years.

1 $$\:\mathrm{S}\mathrm{P}\mathrm{I}\mathrm{i}\mathrm{j}\mathrm{k}=\frac{CHIRPS\mathrm{i}\mathrm{j}\mathrm{k}-CHIRPSi,mean}{CHIRPSi,\sigma\:}$$

In this study, the monthly SPI was derived from daily precipitation data provided by the Climate Hazards Group InfraRed Precipitation with Station data (CHIRPS). The daily values were aggregated into monthly, annually and multi-month precipitation totals, ensuring consistency with standard SPI computation procedures and enabling reliable comparisons across different temporal scales.

#### Agricultural drought assessment methods

##### Normalized difference vegetation index (NDVI)

The NDVI is frequently employed in remote sensing techniques for vegetation analysis^67^. NDVI can be used as a numerical representation of the greenness, density, and condition of vegetation in an area calculated using indicator data^67^. In this study, NDVI values were obtained from mathematical calculations between the red band and the Near-Infrared Radiation (NIR) band. When using Landsat 8 imagery, the red band is band four (light wavelength 640–670 nm), and the NIR band is band five (light wavelength 850–880 nm).

The NDVI makes a distinction between infrared light, which shows that vegetation significantly reflects light portions, and red light, which shows that vegetation absorbs light portions^67^. NDVI values ranged from 1 to 1. In contrast, negative values indicate water bodies^67^. On the other hand, an NDVI value near + 1 indicates dense vegetation areas^67^. The value of NDVI around 0 may not represent green leaves, which could be urbanized areas^43^. The NDVI uses Eq. 2 to estimate the vegetation areas^43^.

2 $$\:\mathrm{N}\mathrm{D}\mathrm{V}\mathrm{I}=\frac{NIR-RED}{NIR+RED}\mathrm{*}100$$

Where NIR—Near-Infrared band. In terms of higher frequencies, vegetation in good health reflects red and near-infrared (NIR) light^43^. The formula results create inducements between the values of 1 and 1^68^. A high NDVI yield is indicated by low reflectance in the near-red and high reflectance in the near-infrared region^68^.

##### Vegetation condition index (VCI)

VCI analyzes current NDVI values with historical records to determine the relative health of vegetation^69^. It is a more accurate indicator than NDVI for tracking water stress situations and vegetation adaptation to the influence^69^. Through the study by^70^, VCI was computed at a spatial resolution of 250 m for the time frame from 2001 to 2022, using data from the MODIS spectroradiometer MOD13Q1 product. The VCI was calculated using Eq. 3^70^ shown below.

3 $$\:\mathrm{V}\mathrm{C}\mathrm{I}=\frac{NDVI-NDVImin}{NDVImax-NDVImin}\mathrm{*}100$$

Where NDVImin and NDVImax denote the historical minimum and highest NDVI values, respectively. The numerator represents the variance between the highest and lowest NDVI values for a given period, and this variation signifies the state of vegetation growth^70^. Vegetation growth decreased when the score was low^70^. The VCI was determined using this formula, yielding a numerical range between 0 and 100^70^. Severe drought was indicated by a VCI value less than 10, moderate drought by a value less than 30, mild drought by a value less than 40, and no drought by a value greater than 40^70^.

##### Land surface temperature (LST)

An important factor in Earth’s energy balance is land surface temperature (LST), which affects how energy is divided into sensible and latent heat fluxes^70^. The integration of vegetation indices with LST is widely applied for drought monitoring and plant stress detection^71^. LST provides valuable information on vegetation health, soil moisture conditions, and the surface–atmosphere interface^72^. A commonly used source for remote sensing–based LST estimates is the Moderate Resolution Imaging Spectroradiometer (MODIS), which generates LST products using thermal bands^73^. MODIS LST products are typically converted into degrees Kelvin using a scaling factor of 0.02^74^. To obtain values in degrees Celsius, an additional adjustment of 273.15 is subtracted from the Kelvin outputs^73^. Then the LST data were rescaled and converted into degrees Celsius (°C) (Eq. 4).

4 $$\:\mathrm{L}\mathrm{S}\mathrm{T}=\left(\epsilon\:*0.02\right)\--273.17$$

##### Temperature condition index (TCI)

The Temperature Condition Index (TCI)^75^ is typically employed to identify temperature-related stress on vegetation, often resulting from excessive moisture in a particular area. This stress can be attributed to the abundance of moisture^75^. With VCI concentrating on moisture circumstances and TCI on temperature conditions, both indicators are useful for evaluating weather-related changes in vegetative health^75^. Nonetheless, VCI has limitations in its ability to detect early-stage drought conditions, specifically prior to biomass degradation, when foliage is still green^70^. On the other hand, LST offers more details on the condition of the vegetation. Plant evapotranspiration increased when LST increased, reducing the amount of available soil moisture. This proves that LST is a reliable measure of vegetative stress, even when plants are still green. A TCI score below 10 indicates severe drought, less than 20 indicates severe drought, less than 30 indicates moderate drought, less than 40 indicates mild drought, and values above 40 indicate no drought^70^. The TCI was calculated using a specific Eq. 5^70^.

5 $$\:\mathrm{T}\mathrm{C}\mathrm{I}=\frac{LSTmax-LST}{LSTmax-LSTmin}$$

Where LST—Land Surface Temperature, LSTmax, LSTmin—Maximum and Minimum respectively.

##### Vegetation health index (VHI)

VHI has grown to be extensively employed to investigate and monitor droughts across the world by combining the results of VCI and TCI by assigning equal weights to their contributions^16^. The VCI and TCI independently measure vegetation moisture and temperature conditions, respectively^23^. Low VCI values indicate vegetative stress caused by drought and insufficient precipitation^70^. By focusing on moisture conditions, VCI helps determine how factors such as soil moisture and precipitation affect vegetation health. Lower temperatures are indicated by higher TCI readings, which may be favorable conditions for vegetation growth^70^. This illustrates how vegetation responds to temperature variations. The combined effects of temperature and moisture on vegetation stress can be evaluated by combining VCI and TCI. The use of both indices allows for a more detailed assessment of how climate change affecting vegetation. The use of both indices allows for a more detailed assessment of how climate change is affecting vegetation. The Vegetation Health Index (VHI), a synthesized metric that incorporates TCI and VCI^69^, acts as an efficient early drought warning system. VHI was evaluated based on the following Eq. 6^69^.

6 $${\mathrm{VHI}}=\alpha *{\mathrm{VCI}}+({\mathrm{1}} - \alpha ){\text{ TCI}}$$

Where α = 0.5 (the contribution of VCI and TCI to the overall vegetation health) and VHI = Vegetation Health Index, VCI = Vegetation Condition Index, and TCI = Thermal Condition Index are used. VHI values range from 0 to 100, where higher values denote healthy vegetation and lower values indicate unhealthy vegetation.

#### The drought hazard index (DHI) calculation

The DHI is a composite index that combines multiple variables to provide a single assessment of the danger and severity of a drought. Uses a combination of agricultural and meteorological drought frequency maps to evaluate droughts. This is important and was applied in various studies^76,77^. By summing the images from each year, the frequency of drought at each pixel level was calculated in over 50%, 30%–50%, or less than 30% of the years, respectively^77^. The frequency map was subsequently classified into five drought risk categories based on the following criteria: 0–1 (no drought risk); 2–6 (low drought risk); 7–11 (moderate drought risk); 12–16 (high drought risk); and ≥ 17 (very high drought risk) zones^23,76,78^. A typical technique is normalizing and weighting individual variables, while the precise formula for the DHI can change based on the methodology and particular indicators employed^79^. Equation 7 was used to obtain the DHI index.

7 $${\mathrm{DHI}}=({\mathrm{VHI}} * {\mathrm{VHInorm}})+({\mathrm{VCI}} * {\mathrm{VCInorm}})+({\mathrm{TCI}} * {\mathrm{TCInorm}}+{\mathrm{SPI}}+{\mathrm{SPInorm}})$$

Where norms are near-normal ratings given to each DI, ѡ are weights assigned according to each DI’s importance.

All maps presented in this study were produced by the authors. The study area map (Fig. 1) was prepared using ArcGIS version 10.4 (Esri, Redlands, CA, USA; https://www.esri.com). Drought indices and their spatial distributions were computed and visualized using the Google Earth Engine (GEE) platform (https://earthengine.google.com). No copyrighted or third-party base maps were used. All datasets were derived from open-access sources and processed under the Creative Commons Attribution (CC BY) license.

**Fig. 2:**
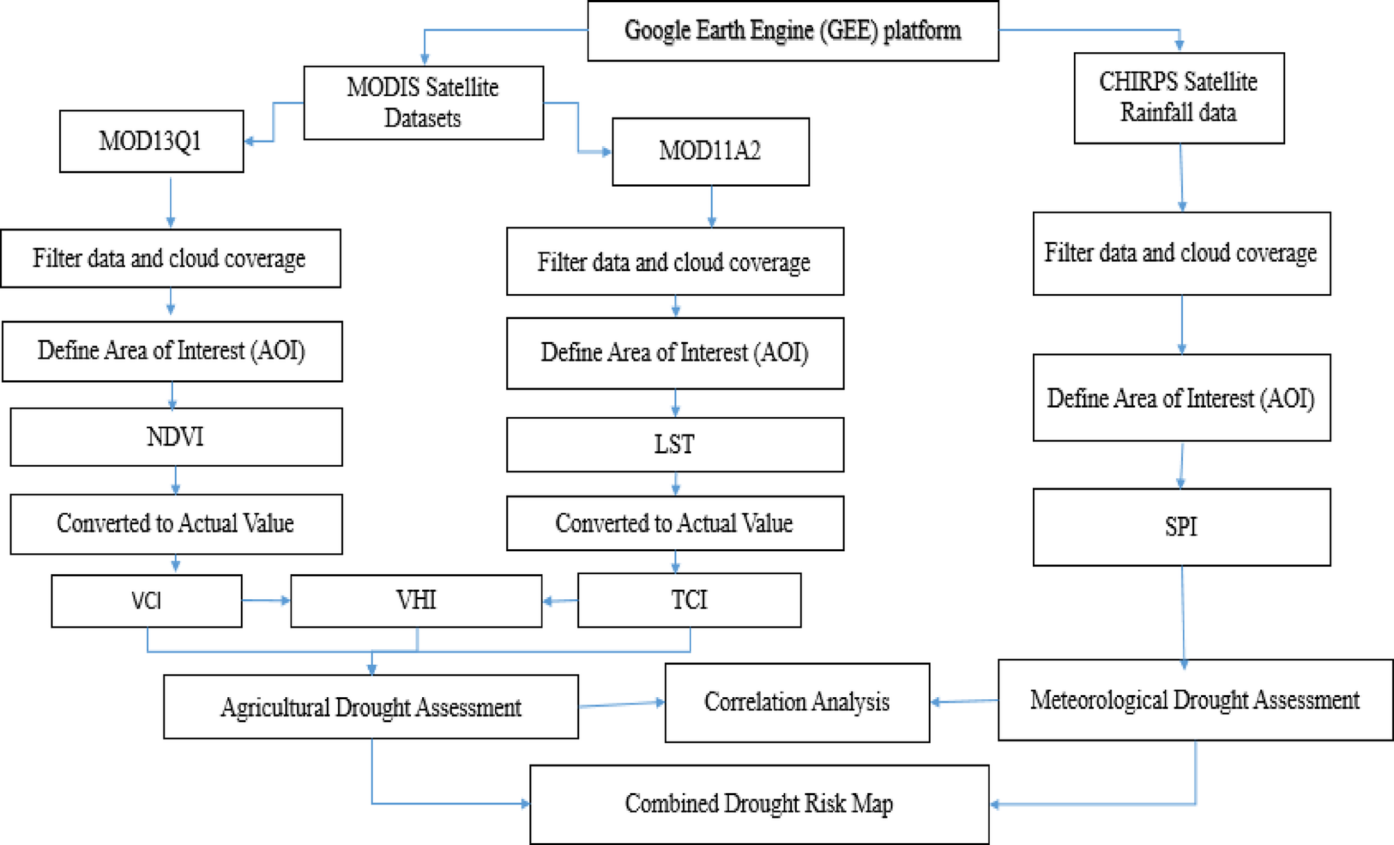
Workflow of the data processing and analysis.

The workflow of this study is illustrated in Fig. 2.

## Results

### Drought analysis

#### Vegetation condition index (VCI)

**Fig. 3:**
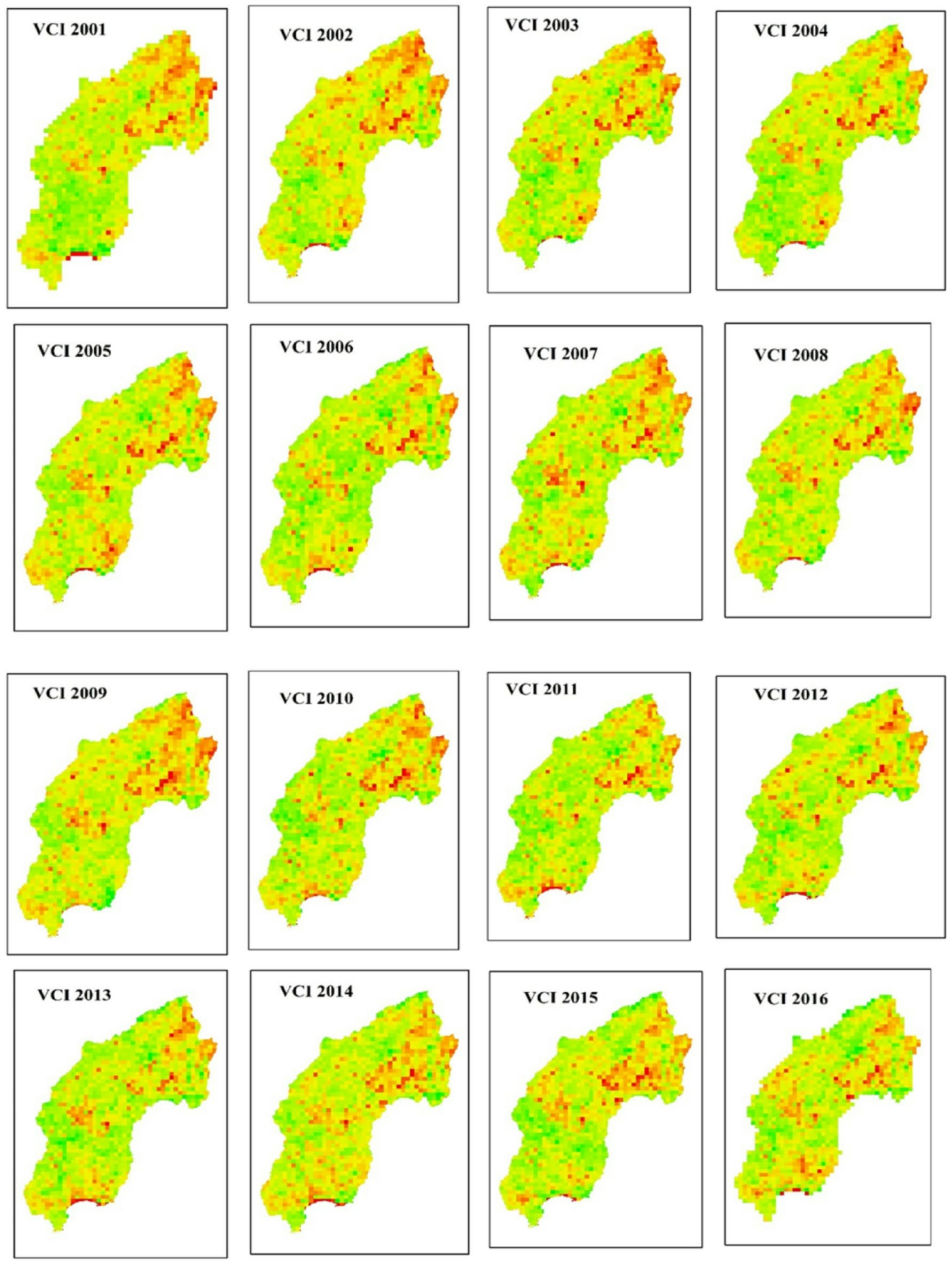

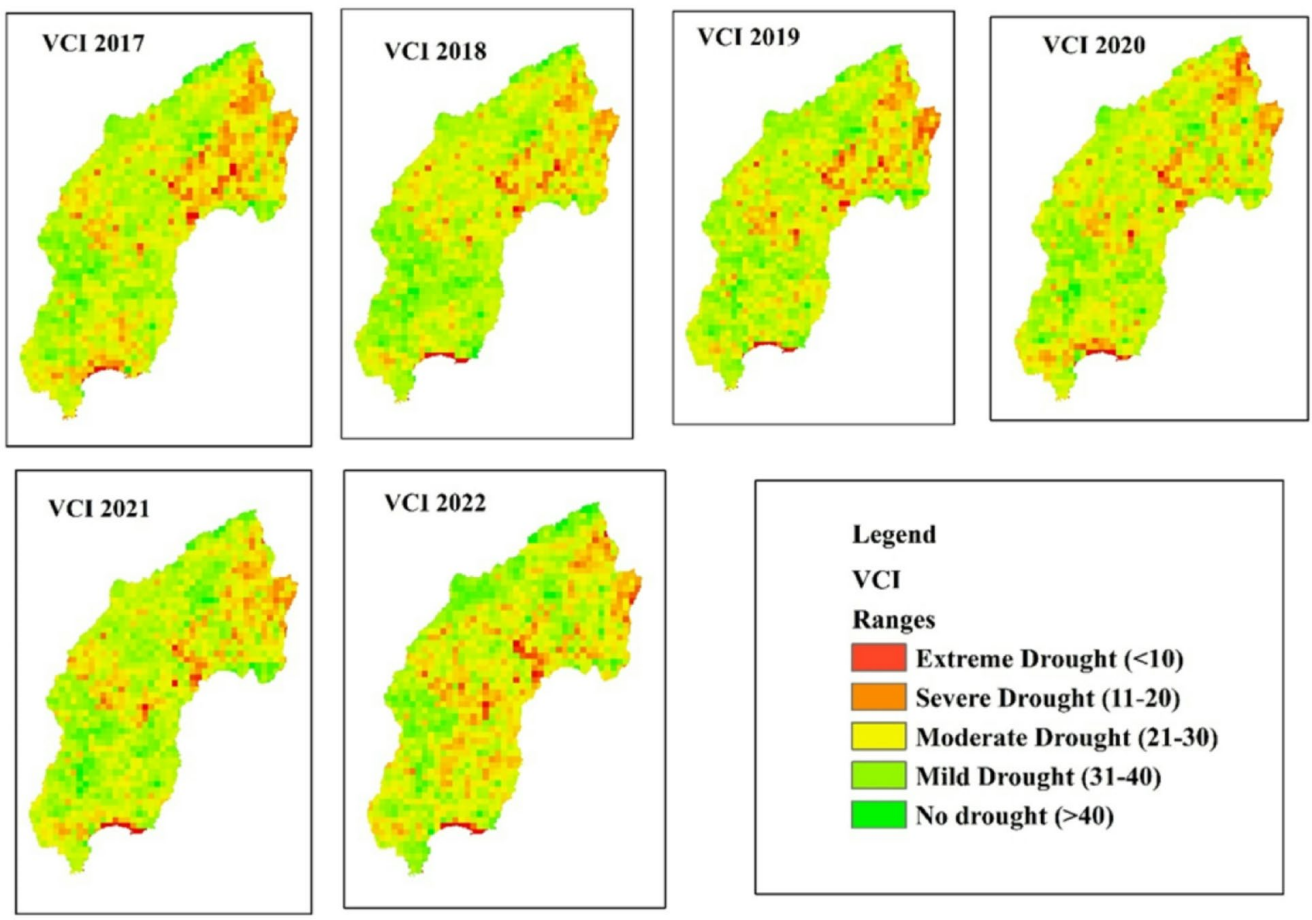
Spatial distribution of drought through VCI during the period 2001–2022.

**Fig. 4:**
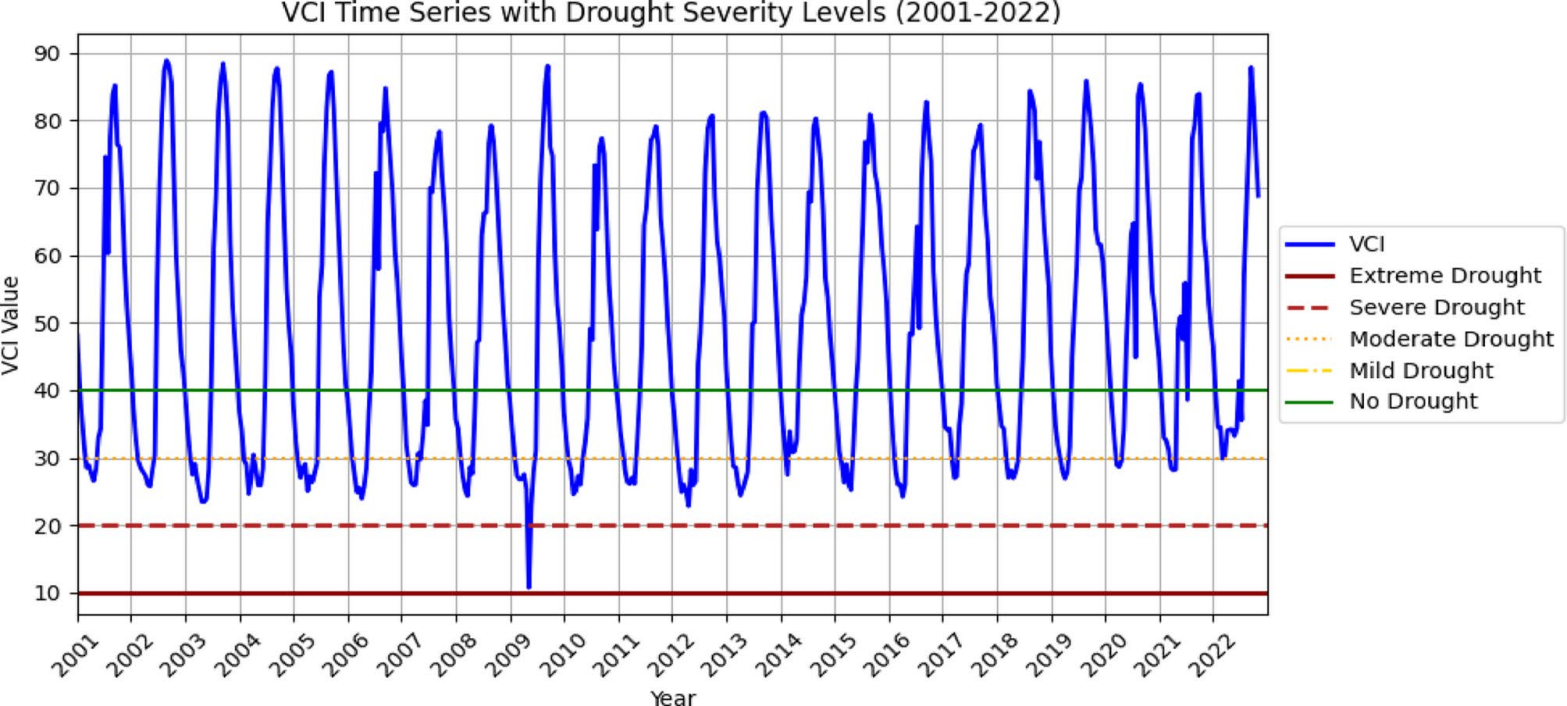
VCI, time series plot of the Megech-Dirma watershed derived using GEE.

The spatial distribution of drought throughout the Megech-Dirma watershed from 2001 to 2022 is shown in Fig. 3, as obtained by VCI. The findings showed that the Megech-Dirma watershed was mostly affected by extreme and severe droughts in the northeast, moderate, and mild droughts in the southwest. The study period’s mean values ranged from 10 to 88, according to the GEE-extracted VCI time-series chart (Fig. 4). The time series revealed minimum VCI values (severe drought) in 2009 and 2010, while moderate, mild, and no drought conditions were observed during 2001–2009 and 2010–2021. In 2009 and 2010, moderate droughts showed a declining trend.

These findings are consistent with previous studies. Enyew et al. who assessed drought in the Menna Watershed, northwestern Ethiopia, also identified 2009, 2011, 2015, 2017, 2019, and 2020 as drought years^16^. Burka et al. reported 2009 and 2015 as severe drought years in the Bilate River watershed, Rift Valley of Ethiopia^27^. Similarly, Senamaw et al. reported 2009 and 2015 as severe drought years in the Waghimara Zone^76^. Furthermore, Tela et al. examining agricultural drought in the Tekeze Watershed (a neighboring area), found that 2004, 2008–2018, 2021, and 2022 experienced mild drought conditions on average^77^. The current results are more strong and reliable due to the overlap in drought years between investigations.

The spatio-temporal analysis further indicated that vegetation cover has gradually improved over the years, reaching mild drought conditions in 2022. However, drought severity remained higher in the northern part of the Megech-Dirma watershed compared to the southern areas.

#### Temperature condition index (TCI)

**Fig. 5:**
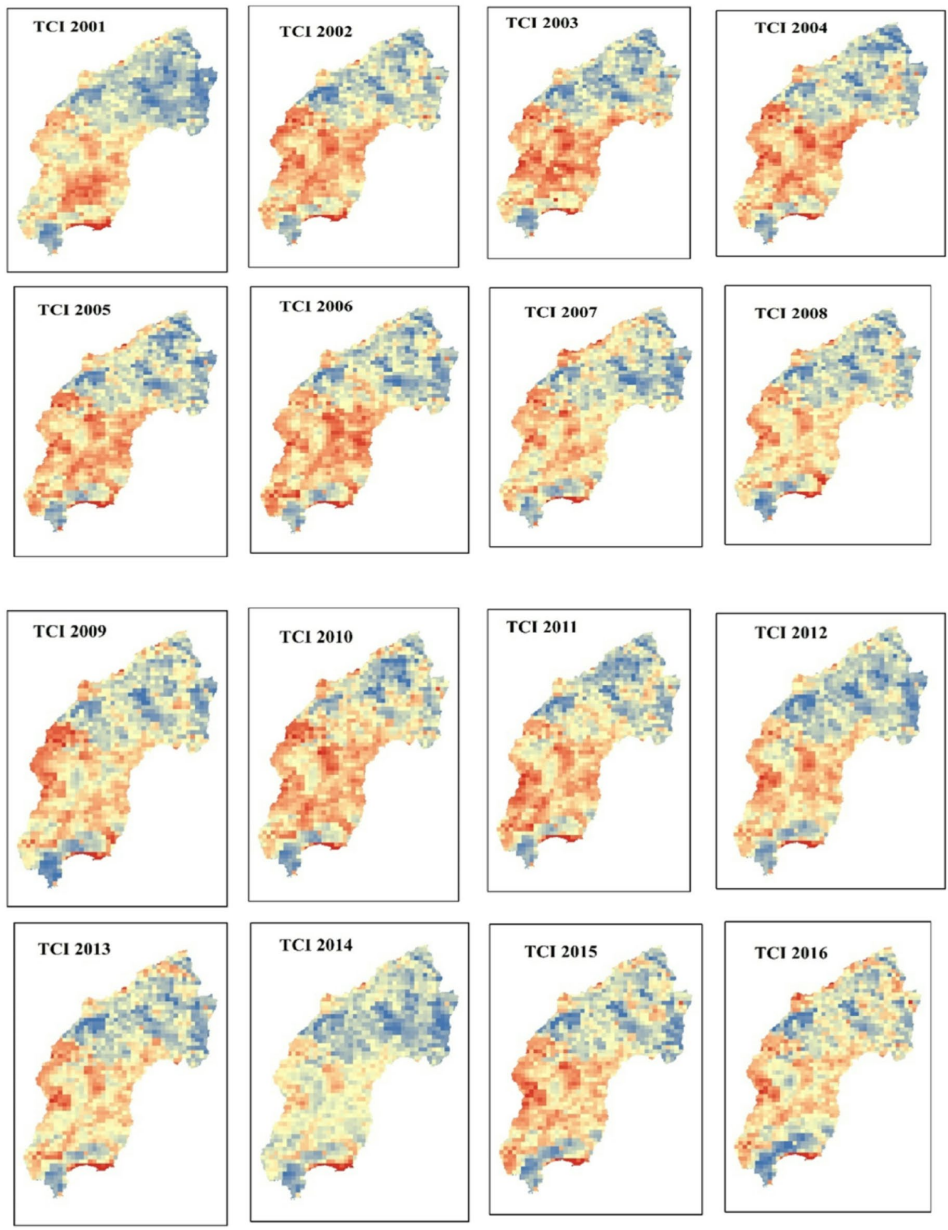

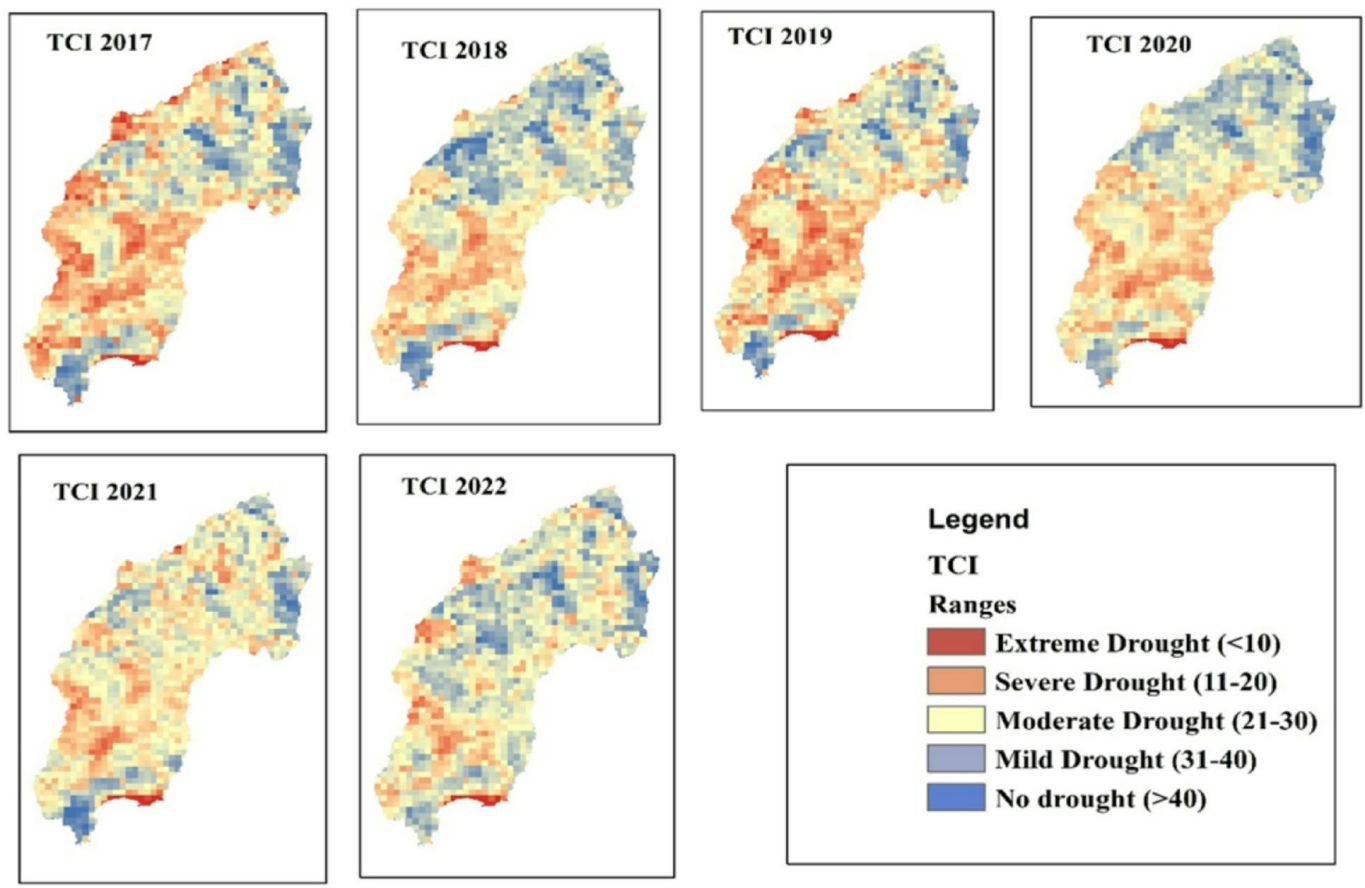
Spatial distribution of drought through TCI during the period 2001–2022.

**Fig. 6:**
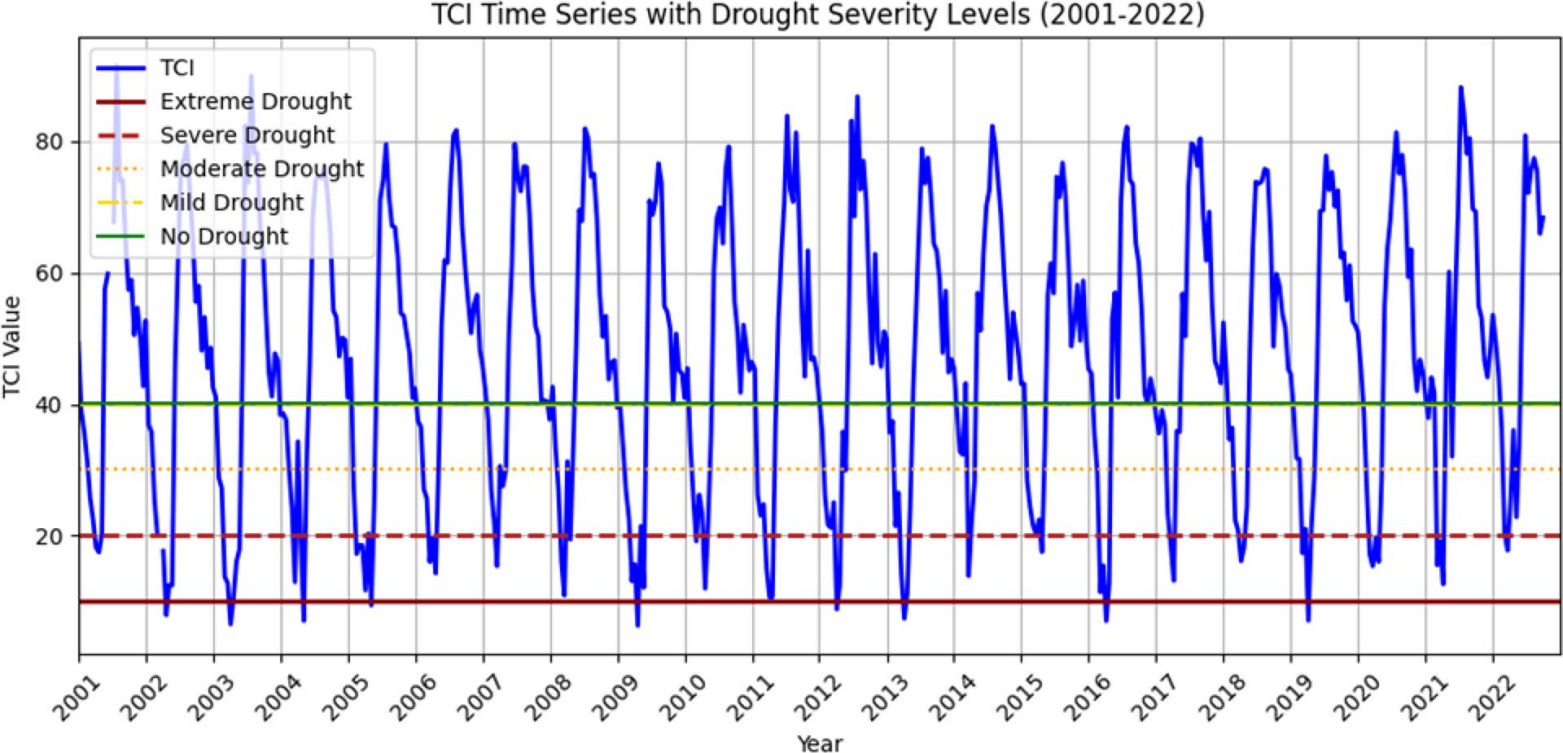
TCI, time series plot of the Megech-Dirma watershed derived using GEE.

The spatial distribution of drought throughout the Megech-Dirma watershed from 2001 to 2022 is shown in Fig. 5, as obtained by TCI. The results showed that the drought conditions are severe and extreme in the southwest and central parts of the watershed, while there are mild drought conditions in the northern part of the Megech-Dirma watershed. The TCI time series revealed frequent extreme drought events in 2002, 2003, 2004, 2009, 2013, 2016, and 2019, while severe drought occurred in 2006, 2007, 2010, 2014, 2014,2015, 2017, 2021, and 2022 (Fig. 6). The years 2001, 2002, 2008, 2018, 2020, and 2022 show mild and moderate drought.

These findings are consistent with previous studies. Enyew et al. assessing drought in the Menna Watershed, northwestern Ethiopia, identified 2002, 2009, and 2015 as highly affected drought years^16^. Similarly, Burka et al. reported 2002, 2009, 2015, and 2022 as severe or extreme drought years in the Bilate River watershed, Rift Valley of Ethiopia^27^. Furthermore, Tela et al. examining agricultural and meteorological drought using geospatial techniques in Ethiopia, found that 2009 and 2015 were drought years^77^. The current results are stronger and more reliable due to the overlap in drought years between investigations.

Overall, mean TCI values across the study area ranged from 6 to 86. Spatial analysis indicated that the southwest part of the Megech-Dirma watershed experienced more severe temperature stress than the northeast, highlighting spatial variability in thermal conditions. Drought intensity and TCI values are inversely correlated; higher drought intensity is associated with lower TCI values.

#### Vegetation health index (VHI)

**Fig. 7:**
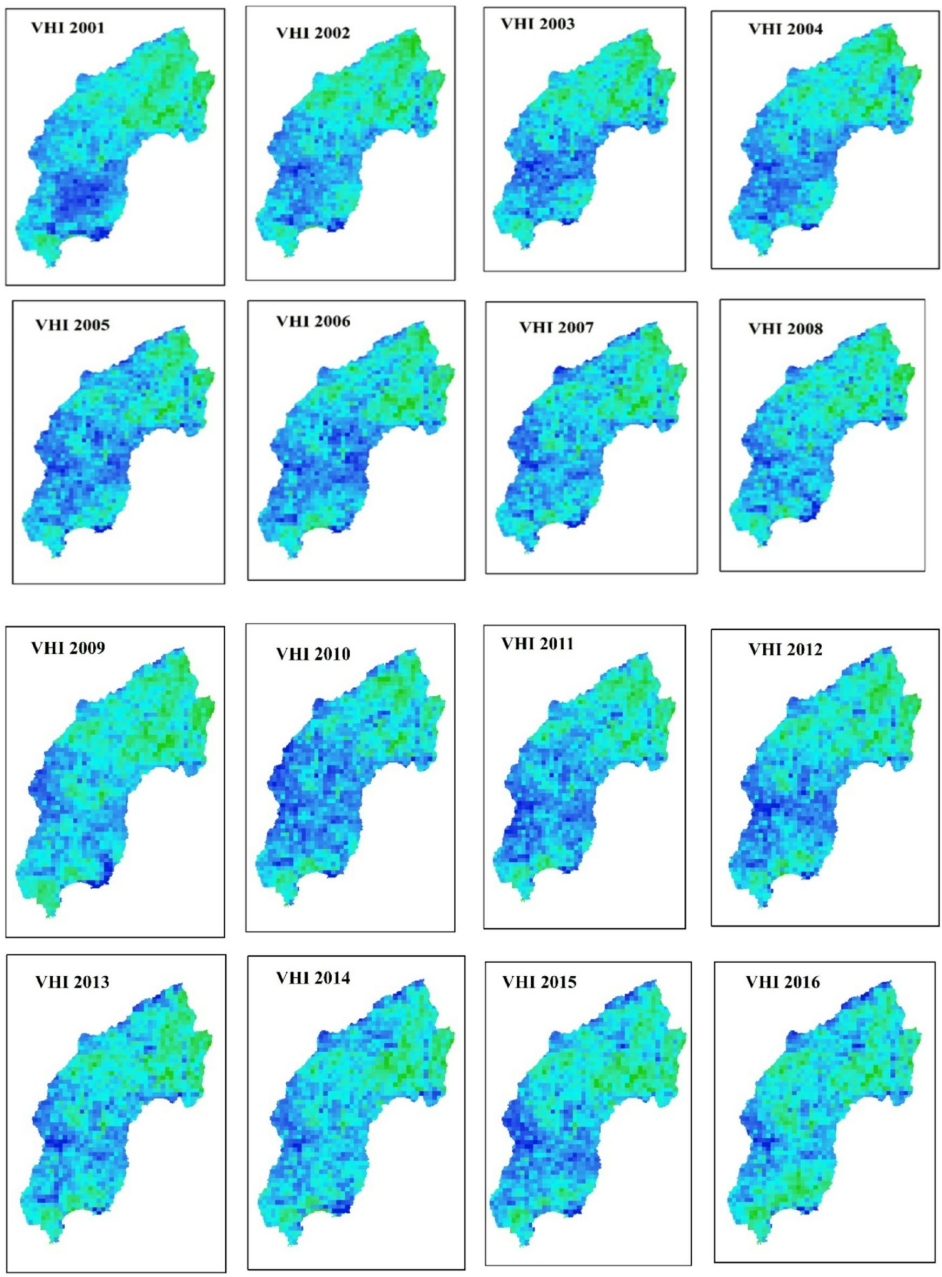

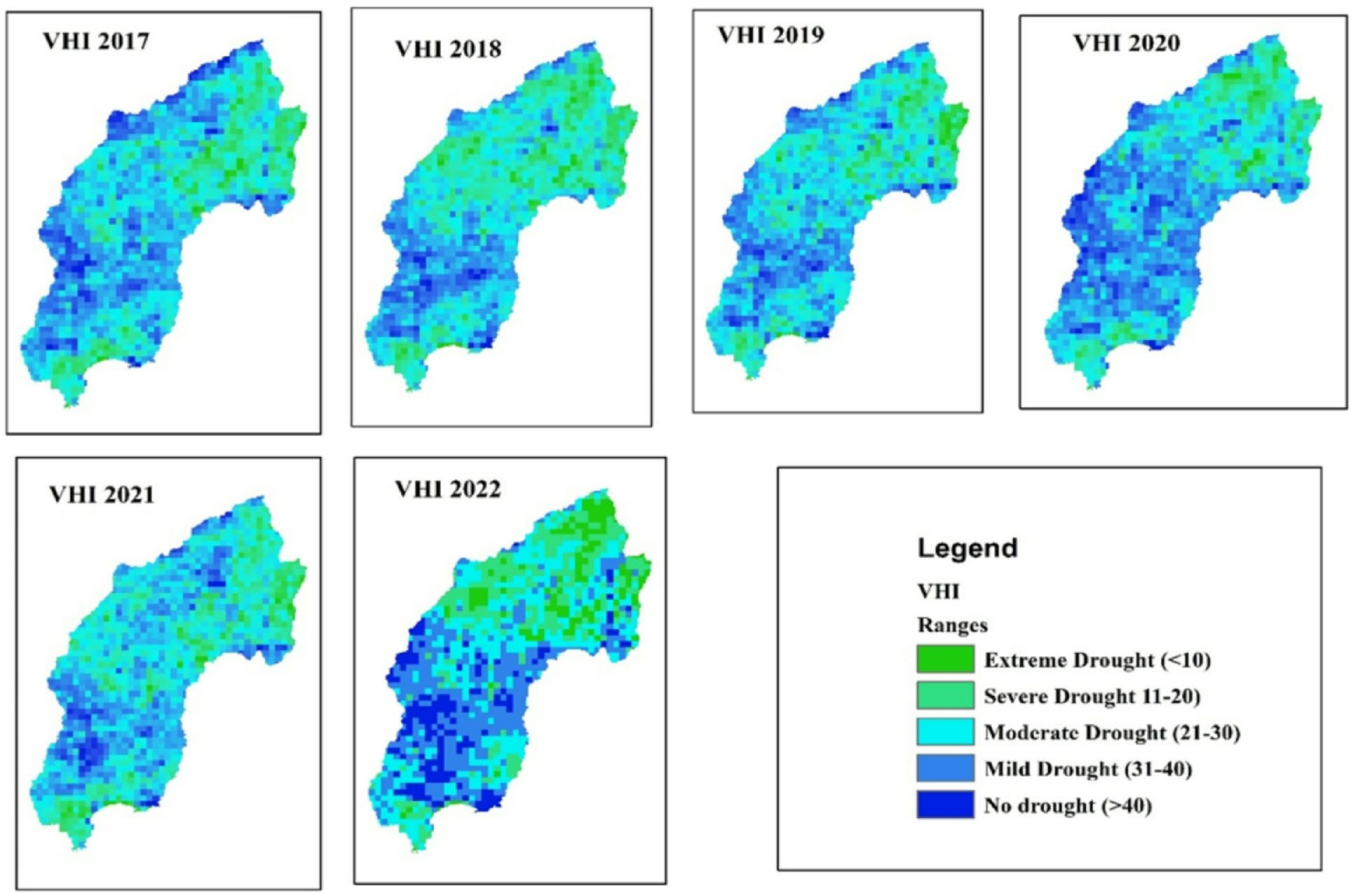
Spatial distribution of drought through VHI during the period 2001–2022.

**Fig. 8:**
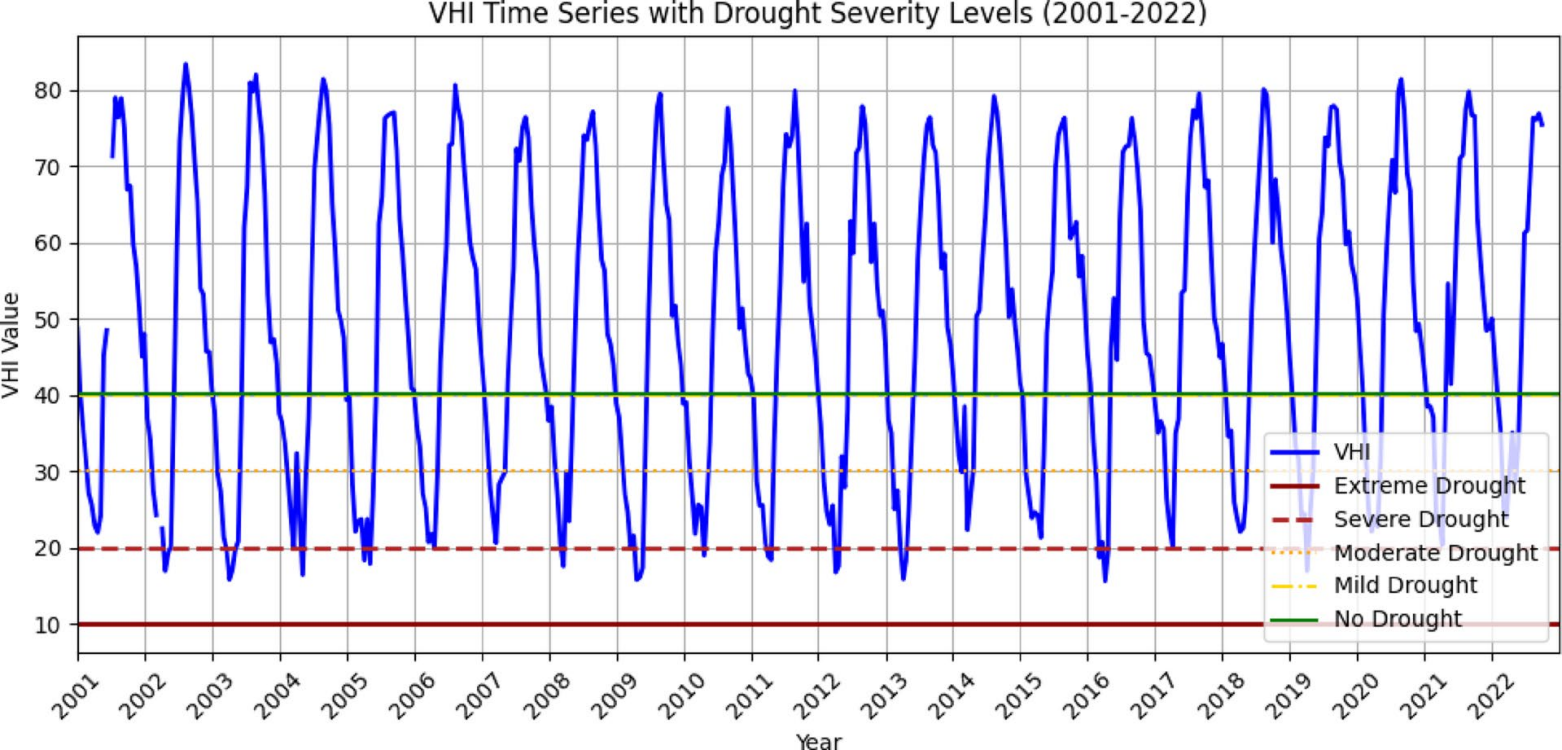
VHI, time series plot of the Megech-Dirma watershed derived using GEE.

The spatial distribution of drought throughout the Megech-Dirma watershed from 2001 to 2022 is shown in Fig. 7, as obtained by VHI. The findings showed that the Megech-Dirma watershed was mostly affected by extreme and severe droughts in the northern part of the watershed and moderate and mild droughts in the southern and central parts of the study area. The GEE-extracted VHI time-series chart showed mean values ranging from 16 to 84 over the study period (Fig. 8). Minimum VHI values, indicating severe drought, occurred in 2002, 2003, 2004, 2009, 2012, 2013, and 2016, while 2001, 2006, 2007, 2010, 2014, 2015, and 2017–2022 were characterized by mild and moderate drought conditions.

These results are consistent with previous studies. Enyew et al. assessing drought in the Menna Watershed, northwestern Ethiopia, identified 2002, 2004, and 2009 as severe agricultural drought years, while 2015 showed moderate drought conditions^16^. Similarly, Burka et al. reported 2002, 2009, 2015, and 2022 as severe or extreme drought years in the Bilate River watershed, Rift Valley of Ethiopia^27^. Furthermore, Tela et al. examining agricultural and meteorological drought using geospatial techniques in Ethiopia, found that 2009 and 2015 were drought years^77^. The current results are stronger and more reliable due to the overlap in drought years between investigations.

The VHI time-series analysis highlights that the Megech-Dirma watershed consistently experienced moderate to mild drought events throughout the study period, with an observable increase in the severity and frequency of moderate droughts. Comparatively, VHI provided a more comprehensive assessment than TCI or VCI, as it integrates vegetation vigor and thermal stress. Previous studies^27,76,80^ also confirm the effectiveness of VHI for drought detection in similar regions.

#### Standardized precipitation index (SPI)

**Fig. 9:**
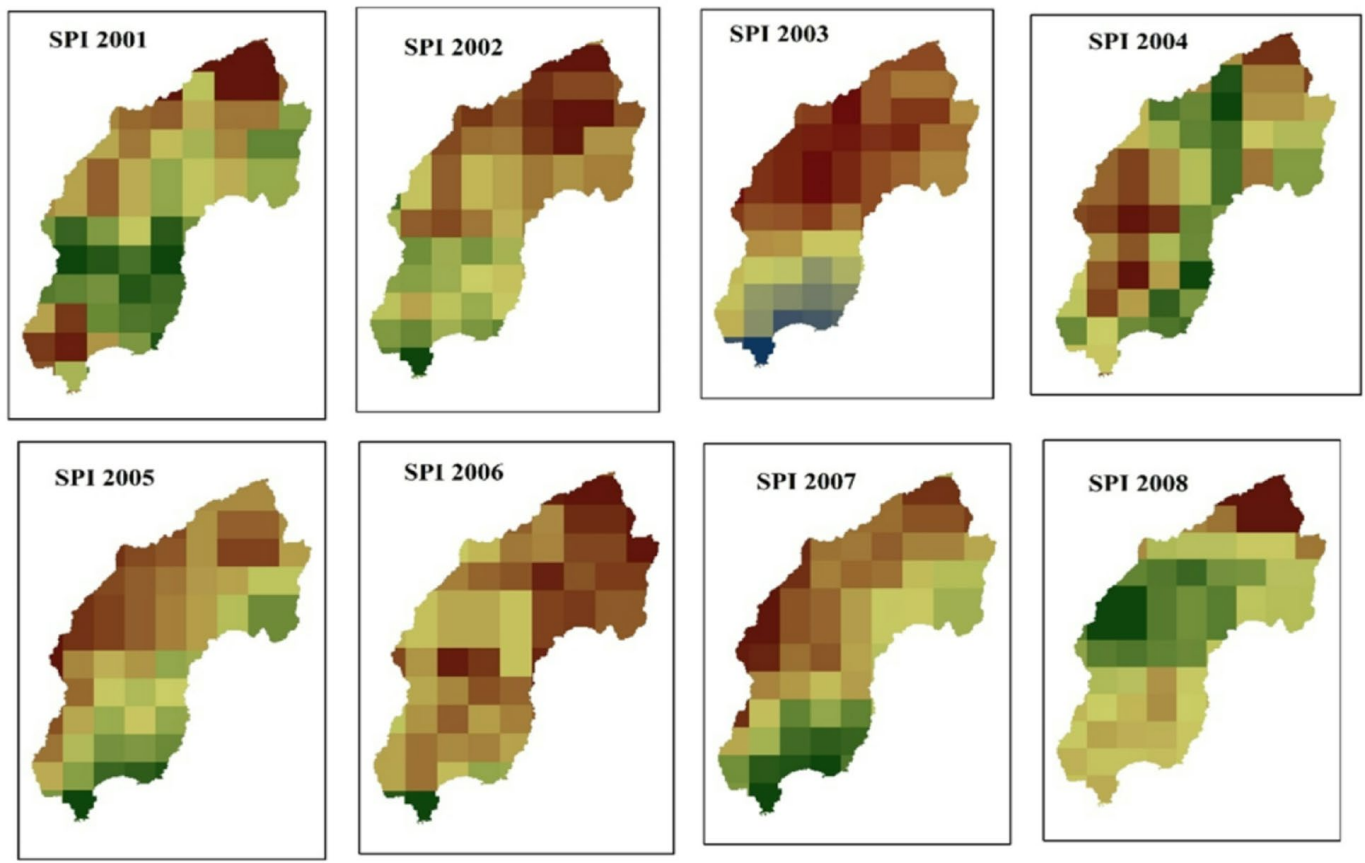

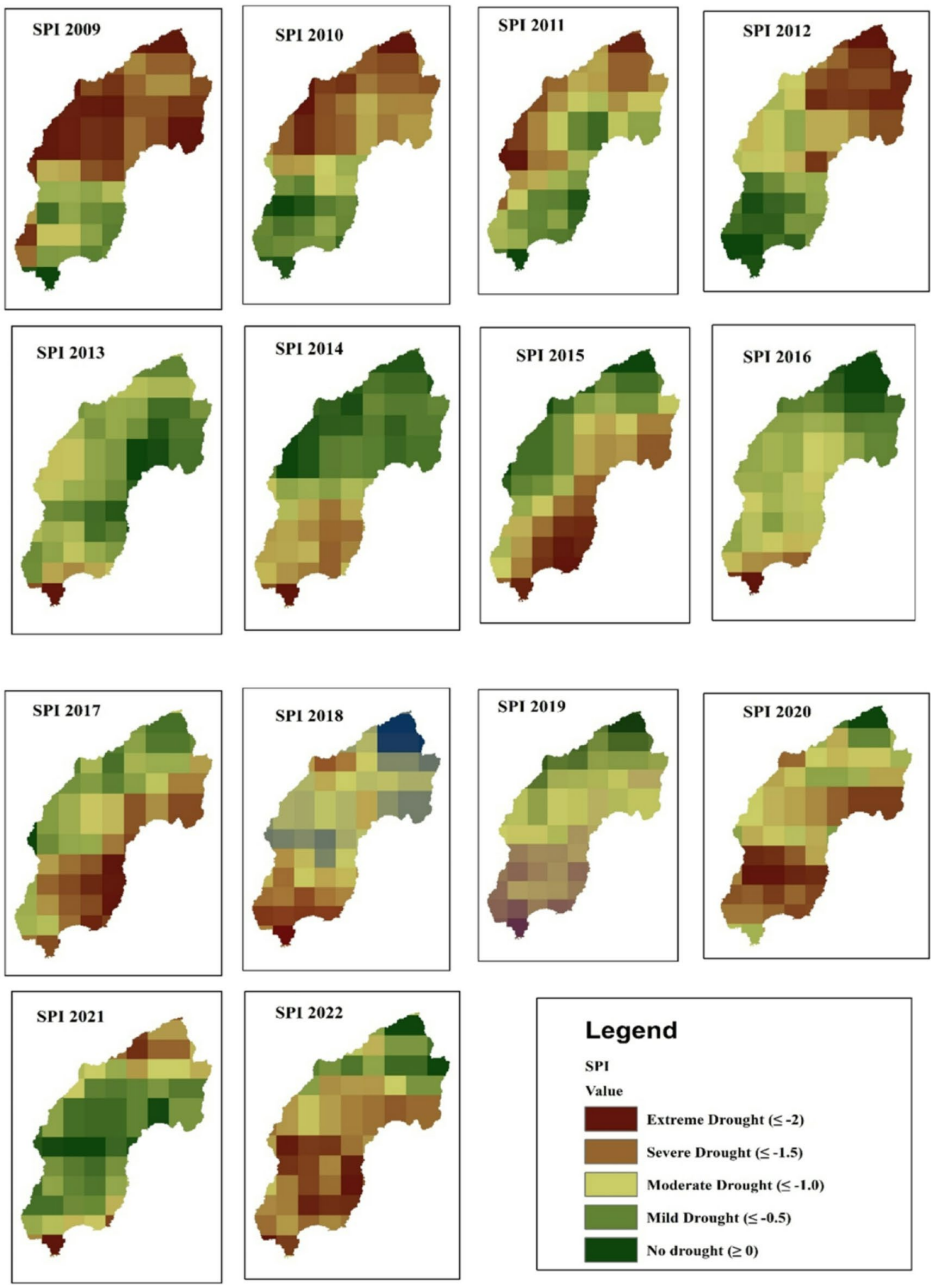
Spatial distribution of drought through SPI during the period 2001–2022.

**Fig. 10:**
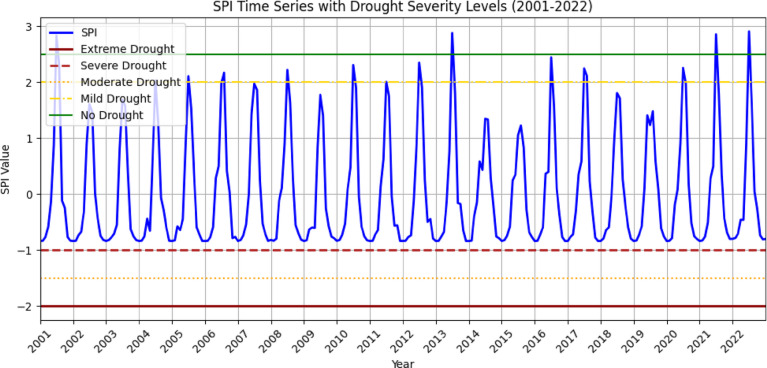
SPI, time series plot of the Megech-Dirma watershed derived using GEE.

The spatial distribution of drought throughout the Megech-Dirma watershed from 2001 to 2022 is shown in Fig. 9, as obtained by SPI. Spatial analysis revealed that severe and extreme drought conditions were distributed across the northern, central, southern, and middle-eastern regions of the watershed, while the moderate drought was distributed in the southeastern and northeastern regions of the watershed. The GEE-extracted SPI time-series chart showed mean values ranging from − 1 to 3 over the study period (Fig. 10).

These results are consistent with previous studies^21^. , considering annual SPI under the RCP4.5 scenario, reported that extreme meteorological drought is likely in the Gumara and Ribb watersheds, while moderate drought occurs in Megech and Gilgel Abbay. Similarly, Senamaw et al. found that northeastern Ethiopia is frequently affected by meteorological drought based on SPI analysis^76^. The drought years identified by SPI in the Megech-Dirma watershed correspond to periods of substantial economic and ecological impact, consistent with findings from other Ethiopian watersheds^21,54,76^.

The reliability of the CHIRPS rainfall dataset used in this study was validated through previously published work. Enyew and Wassie^54^ compared CHIRPSv2 rainfall with ground-based observations from seven stations (Ambagiorgis, Dabat, Enfraz, Gondar, Makisegne, Debark, and Tikil Dingay) across seasonal and annual timescales. Their results demonstrated strong agreement, with annual correlation coefficients ranging from 0.75 at Makisegne to 0.88 at Gondar. Similarly, Alemu and Bawoke^81^, working in the Amhara region, reported coefficients of determination (R²) between 0.89 and 0.93 across several gauging stations, confirming CHIRPS as a reliable proxy for observed rainfall. Comparable findings were also reported by Wassie et al. in North Wollo (R² = 0.82)^23^, and Bayisa et al. in the upper Blue Nile basin, Ethiopia (R² = 0.86)^82^. Collectively, these studies establish CHIRPS rainfall data as a robust and effective tool for drought detection and assessment, particularly in regions where ground-based data are limited. More broadly, several national and international studies have recommended CHIRPS for regional climate and drought studies, both in Ethiopia^16,23^ and globally^53,62^. Such consistent validation confirms CHIRPSv2 as a reliable dataset for monitoring meteorological drought in data-scarce regions like the Megech-Dirma watershed.

### Correlation coefficient analysis between drought indices (DIs)

**Fig. 11:**
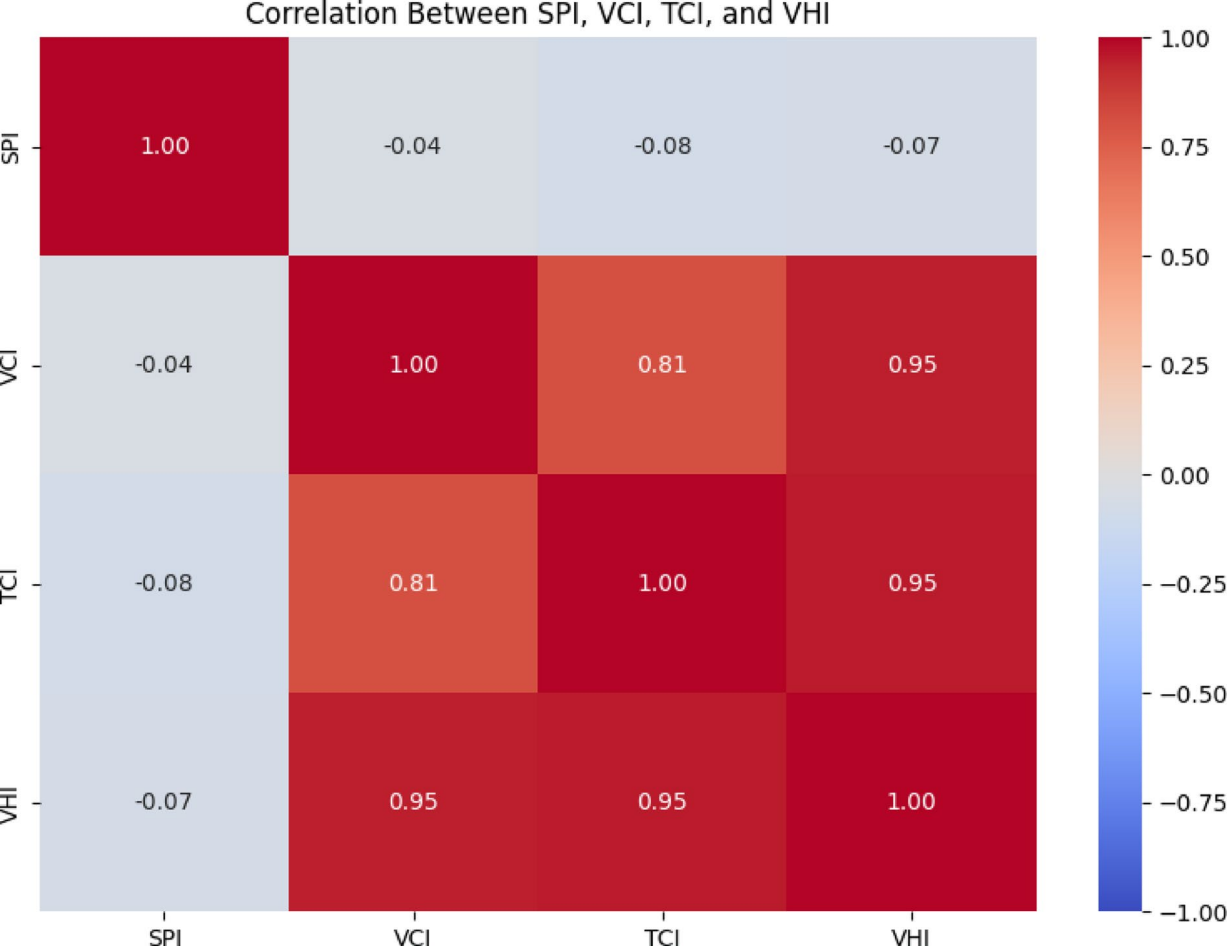
Correlation heat map of drought indices (DIs).

Correlation matrices were employed to determine whether the features were related to one another. A positive value indicates a positive correlation when the variables move in the same direction, whereas a negative value indicates a negative correlation. The correlation analysis between the DIs (TCI, VCI, VHI, and SPI) across the study period is displayed in Table 1; Fig. 11. With a correlation coefficient of 0.95, the VHI index showed the strongest positive association with the VCI and TCI. The SPI index showed a weak correlation with the VCI, TCI, and VHI indices, with correlation coefficients of -0.04, -0.08, and − 0.07, respectively. Compared to the VHI with VCI and TCI, the correlation between the VCI and TCI indices was less strong. The correlation between SPI and vegetation-based indices was weak in our study due to several reasons. First, SPI measures only rainfall anomalies, whereas VCI, TCI, and VHI capture vegetation responses that are influenced by multiple factors, including soil moisture storage, evapotranspiration, and temperature extremes. Second, vegetation stress often reflects cumulative or lagged rainfall effects rather than immediate precipitation anomalies, which weakens the contemporaneous correlation. Third, spatial mismatches exist between rainfall datasets (CHIRPS) and high-resolution MODIS vegetation indices, further reducing correlation strength. Finally, land use practices (e.g., irrigation or crop type) may decouple vegetation response from rainfall variability. For these reasons, the weak correlation values (-0.08 to -0.06) observed in our study are consistent with findings in other drought assessments, where meteorological and agricultural indices often diverge.

**Table 1:** Correlation coefficient analysis of drought indices (DIs).

	SPI	VCI	TCI	VHI
SPI	1	− 0.04	− 0.08	− 0. 07
VCI	− 0.042	1	0.81	0.95
TCI	− 0.081	0.81	1	0.95
VHI	− 0.07	0.95	0.95	1

### Combined drought risk map

**Fig. 12:**
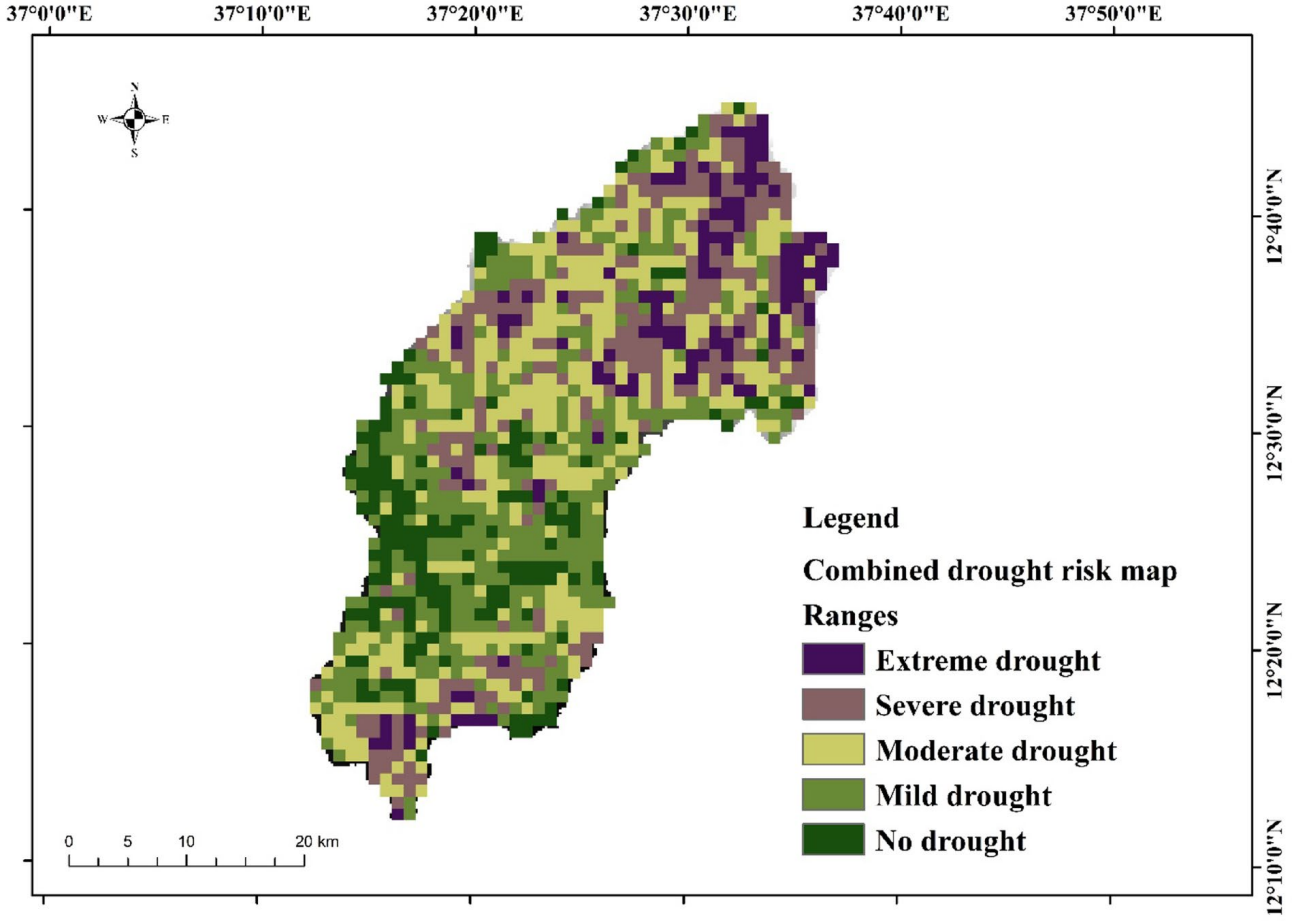
Combined drought risk map in the Megech-Dirma watershed.

The final drought risk map was developed by overlaying agricultural and meteorological drought maps. Weights were assigned using pairwise comparison, giving 60% weight to agricultural drought due to its higher influence on local livelihoods. The resulting drought hazard maps (Fig. 12) show that drought severity was extreme drought and severe drought in the northern and southern parts of the study area, while moderate drought, mild drought, and no drought were located in the southwest, eastern, and central parts of the Megech-Dirma watershed.

The area coverage of each drought class is summarized in Table 2: extreme drought (9.63%), severe drought (20.95%), moderate drought (29.12%), mild drought (26.94%), and no drought (13.36%). These findings are in line with, Wubneh et al. who reported that large parts of the Lake Tana sub-basin—proximate to the Megech-Dirma watershed—are highly prone to moderate drought events^21^.

**Table 2:** Drought hazard severity index.

DHI	Area	Percentage
Extreme drought	123.31	9.63
Severe drought	268.36	20.95
Moderate drought	373.01	29.12
Mild drought	345.07	26.94
No drought	171.08	13.36

## Discussion

Drought disasters exacerbate climatic variability and accelerate land degradation, in addition to significantly affecting human lives, economic development, and agricultural productivity. This study analyzed multiple drought indices—Vegetation Condition Index (VCI), Temperature Condition Index (TCI), Vegetation Health Index (VHI), and the meteorological Standardized Precipitation Index (SPI)—in the Megech-Dirma watershed from 2001 to 2022.

Evaluating in situ SPI against remotely sensed drought indices (DIs) in the watershed is challenging due to the limited availability of climate data and the sparse distribution of weather stations. Existing records are often incomplete, with missing observations and uncertainties, making it difficult to fully capture precipitation, temperature, and evapotranspiration variability. Despite these constraints, SPI and satellite-derived indices have been widely validated across Ethiopia and East Africa, consistently demonstrating good performance for rainfall and drought monitoring^16,23,27^. Based on this established reliability, we employed SPI and DIs as the most suitable datasets for our analysis. Future research will integrate station-based validation to further strengthen the robustness of our findings.

This approach is supported by prior studies emphasizing the value of integrating multiple drought indicators. Thilagaraj et al. and Al Nadabi et al. demonstrated that combining indices provides a more nuanced understanding of drought severity and its ecological impacts^40,53^. Similarly, Enyew et al. utilized geospatial technologies to monitor agricultural drought and examine climatic extreme indices in the Menna Watershed in northwest Ethiopia^16^. Senamaw et al. in mapping drought-vulnerable areas, further recommended integrating socio-economic data to capture other critical vulnerability factors^76^. Wubneh et al. conducted trend analysis and hydro-meteorological drought forecasts for ungauged basins in the Upper Blue Nile Basin under future climate change scenarios^21^. In line with these studies, our strategy offers a more comprehensive perspective on drought dynamics, which is essential for producing precise and reliable assessments and for informing effective drought management and mitigation strategies^53^.

A key contribution of this study is the correlation and evaluation of DIs (VCI, TCI, VHI, and SPI) from 2001 to 2022 using the Google Earth Engine (GEE) platform and MODIS satellite datasets. This integration enabled long-term and large-scale drought monitoring, leveraging both the wide temporal–spatial coverage of MODIS and the computational capacity of GEE. Similar approaches have proven effective in enhancing drought monitoring accuracy and in capturing spatiotemporal drought dynamics^53^.

However, one of the persistent challenges in agricultural drought assessment in the Megech-Dirma watershed is the scarcity of reliable in-situ climate data, particularly for temperature, which is critical for drought research. The limited spatial coverage and temporal gaps in weather station data restrict the capacity for comprehensive drought assessment^53^. This limitation underscores the importance of remote sensing datasets such as MODIS, which provide consistent, spatially extensive, and continuous coverage. When combined with ground observations, remote sensing data help overcome these challenges, offering a more complete and accurate representation of climate variability and drought dynamics^27^.

Overall, the use of DIs offers valuable insights into the severity, frequency, and progression of drought events. Such insights are essential for identifying patterns and trends, which in turn support the development of effective adaptation and mitigation strategies. The findings of our study are follows:

1. The TCI values across the study period showed considerable fluctuations, with certain years—2002, 2003, 2004, 2009, 2013, 2016, and 2019—recording the lowest values. These correspond to periods when the study area experienced more extreme weather conditions. Compared with 2001–2013, the TCI trend indicates that temperatures increased during 2014–2022, reflecting a worsening of temperature stress that may have significant ecological and agricultural implications for the region.
  The mean TCI values varied from 6 to 86 across different years, underscoring the spatial and temporal variability of drought severity. This highlights the importance of considering multiple indices, such as TCI and VCI, when evaluating drought dynamics^53^. Burka, et al.^27^ also examined drought distribution and dynamics in the Bilate River watershed (Rift Valley, Ethiopia) using the Google Earth Engine platform with TCI, VHI, and VCI. Their findings similarly demonstrated the role of low TCI values in identifying periods of extreme temperature stress and their effects on agriculture. Likewise, Tela, et al.^77^, assessing agricultural and meteorological drought using geospatial techniques in Ethiopia, identified 2009 and 2015 as drought years.
  The consistency of drought years across different studies enhances the robustness and reliability of the present findings. Overall, the TCI results emphasize the critical role of temperature variability in shaping drought dynamics and its cascading impacts on ecosystems and livelihoods in the Megech-Dirma watershed.
2. The spatial distribution maps of VCI revealed that moderate drought conditions prevailed during several years between 2001 and 2022, with mean values ranging from 10 to 88. These variations reflect the changing vegetation health and moisture availability in the watershed over time. The results highlight a variable pattern of drought severity, with some years showing relatively wetter conditions while others experienced mild to moderate drought.
  Comparable findings were reported by Burka, et al.^27^, who analyzed spatiotemporal variations of spring drought in the Bilate River watershed in Ethiopia’s Rift Valley. Their results, consistent with the present study, emphasized the value of VCI in capturing vegetation responses to drought stress. Similarly, Niyonsenga, et al.^83^, using statistical models to analyze VCI trends across the Horn of Africa, demonstrated a strong correlation between seasonal rainfall patterns and VCI values. They observed that mean VCI decreased by as much as 20 points during drought years compared to non-drought years and that periods with VCI below 50 corresponded to severe drought events and significant declines in vegetation health. Together, these findings underscore the effectiveness of VCI as a reliable indicator of vegetation stress and moisture availability, providing essential insights into the dynamics of drought in the Megech-Dirma watershed.
3. The VHI spatial distribution maps identified 2002, 2003, 2004, 2009, 2012, 2013, and 2016 as severe drought years, with mean values ranging from 16 to 84 across the study period. These results underscore the persistent nature of drought conditions in the Megech-Dirma watershed and reveal an increasing frequency of severe droughts in years that are more recent.
  Liou and Mulualem^84^ similarly observed a discernible rise in severe drought events in Ethiopia, reinforcing the present study’s findings. The spatial and temporal analysis of VHI provides valuable insight into changing drought patterns, highlighting the need for targeted mitigation and adaptation strategies to reduce vulnerability in the study region.
4. The SPI spatial distribution maps revealed that several years between 2001 and 2022 were marked by moderate drought conditions, with mean values ranging from − 1 to 3 across the study period. The extreme SPI values occurred at the northern tip of the watershed, while severe drought conditions were more widespread in the southern, central, and western regions. In contrast, much of the area exhibited moderate drought patterns, indicating spatial variability in precipitation deficits. Prolonged persistence of such conditions could significantly impact the subsurface hydrological system, particularly groundwater resources, thereby exacerbating water stress in the region^40^. These findings highlight the importance of integrating SPI-based assessments with other drought indices to better capture the multi-dimensional nature of drought impacts.
5. Correlation analyses between the Standardized Precipitation Index (SPI), VHI, TCI, and VCI revealed weak associations between SPI and vegetation-based indices (SPI/VCI, SPI/VHI, SPI/TCI), while correlations among VHI, TCI, and VCI were consistently strong. Although most agriculture in the watershed is rainfed, meteorological and agricultural droughts do not always occur simultaneously. Vegetation response often lags behind rainfall recovery, and stress can persist even when SPI indicates near-normal conditions (SPI > -1). High land surface temperatures, residual soil moisture deficits, and cumulative effects of previous dry periods frequently influence vegetation condition, pulling VHI toward moderate or severe drought categories.
  Moreover, factors such as land use/land cover change, soil degradation, and irrigation limitations further exacerbate vegetation stress independently of rainfall. Agricultural drought is strongly governed by soil moisture dynamics, which are influenced not only by current precipitation but also by soil type, evapotranspiration, and atmospheric temperature. These complexities explain why vegetation-based indices (VCI, TCI, and VHI) may diverge from meteorological indices like SPI, highlighting the need to use both in drought assessment.
  Similar findings have been reported elsewhere. Ejaz et al. found a very strong link between VHI and VCI/TCI in Northern Ethiopia^27^, while Wassie et al. highlighted a significant relationship between VCI and VHI in Menna Watershed, northwestern Ethiopia for monitoring agricultural drought. Similarly, Bayable et al. studying the Upper Awash Basin in Ethiopia, confirmed a strong association between VHI, TCI, and VCI^85^. Together, these findings emphasize that meteorological and agricultural droughts represent related but distinct processes. Recognizing their divergence is essential for effective drought monitoring, agricultural planning, and water resource management.
6. The spatial distribution maps of the Drought Hazard Index (DHI) indicate that the northern regions experienced the highest levels of drought risk, with severity gradually decreasing toward the central and eastern parts of the study area. Specifically, the results showed that extreme drought (9.63%), severe drought (20.95%), moderate drought (29.12%), mild drought (26.94%), and no drought (13.36%). This spatial disparity underscores the importance of recognizing regional differences in drought severity and the necessity of implementing proactive strategies to enhance resilience and reduce impacts on ecosystems and vulnerable communities^53^.

Despite the advances offered by satellite-based drought indices (DIs), several limitations remain. First, ground validation is essential for ensuring accuracy under local conditions, yet this is often difficult in remote or arid regions with sparse monitoring networks. Second, the potential for misinterpretation of satellite-derived indices requires expertise in both remote sensing and climate science; otherwise, assessments of drought severity may be biased. Third, the relatively short temporal coverage of continuous satellite observations (e.g., MODIS data available only since 2001) restricts long-term historical analyses. Fourth, the moderate spatial resolution of satellite imagery may fail to capture fine-scale variations, particularly in heterogeneous landscapes or small-scale agricultural systems. Additionally, while platforms such as Google Earth Engine (GEE) provide access to large and valuable datasets, researchers in resource-constrained settings may face challenges in data handling and processing. Finally, although indices such as VCI, TCI, VHI, SPI, and DHI yield robust quantitative assessments, they may not fully reflect the socioeconomic consequences of drought on local populations—a critical dimension for comprehensive drought risk assessment and management.

## Conclusions

This study assessed the spatial and temporal distribution of droughts, including meteorological droughts estimated through SPI and remote sensing-retrieved drought indices (RSDIs, including VCI, TCI, and VHI). This study contributes valuable information for understanding the spatial and temporal distribution of agricultural droughts in the Megech-Dirma Watershed, with the following conclusions:

1. Throughout the study period, the TCI showed temporal fluctuations, with some years (2002, 2003, 2004, 2009, 2013, 2016, and 2019) showing particularly low values. Furthermore, there was a discernible trend of increasing temperatures from 2014 to 2022 compared to earlier years, indicating the potential impact of climate change.
2. The time series revealed minimum VCI values (severe drought) in 2009 and 2010, while moderate, mild, and no drought conditions were observed during 2001–2009 and 2010–2021, and the mean VCI values ranged from 10 to 88 throughout the study period. This implies that the area experiences sporadic, but mostly mild, vegetation stress.
3. The years 2002, 2003, 2004, 2009, 2013, and 2016 were categorized as severe drought years based on the VHI spatial distribution maps, and the mean VHI values over the study period varied from 16 to 84. A noticeable increase in severe drought events was observed in the later years of the study period, emphasizing the evolving nature of drought patterns and the need for effective mitigation and adaptation strategies.
4. The VHI, TCI, and VCI indices showed a strong positive correlation with one another, implying a strong linear connection and emphasizing the importance of VCI and TCI as predictors of VHI. Although they varied in strength, positive correlations were also found between the VCI and TCI indices, but there was little correlation between the SPI and the VCI, TCI, and VHI indices.
5. According to the SPI results, a considerable number of severe and extreme drought occurrences occurred in 2002, 2007, 2009, 2012, 2015, and 2019. Overall, the findings demonstrated that regional climate change caused notable regional differences in drought intensity.
6. The northern regions of the study area faced the most severe drought hazards, gradually diminishing towards the south and east. Approximately 29.12% of the total area was classified as under moderate drought risk, and 9.63% faced extreme drought risk, underscoring the widespread and significant impacts of drought in the study area.

This study offers insights for local experts to monitor drought in near real-time, as it is the first to employ GEE for drought monitoring in Ethiopia’s Megech-Dirma watershed. The study also demonstrated that GEE is a useful tool for drought monitoring at the watershed level. To ascertain the area’s susceptibility to the consequences of drought, more research in the watershed should be conducted, taking into account additional drought-causing and aggravating elements such as socioeconomic and other biophysical data.

## Future work

We plan to improve our methodology by (i) incorporating available ground station data for validation, (ii) integrating higher-resolution datasets, when possible, (iii) exploring machine learning downscaling approaches, and (iv) linking satellite-based drought indicators with socio-economic datasets for more comprehensive assessments.

## Data Availability

The datasets generated and/or analyzed during the current study are available from the corresponding author upon reasonable request.
